# Evaluation of common bean (*Phaseolus vulgaris*) genotypes for resistance to common bacterial blight and angular leaf spot diseases, and agronomic performances

**DOI:** 10.1016/j.heliyon.2022.e10425

**Published:** 2022-08-28

**Authors:** Fekede Girma, Chemeda Fininsa, Habtamu Terefe, Berhanu Amsalu

**Affiliations:** a Department of Plant Sciences, Dilla University, P.O. Box 419, Dilla, Ethiopia; b School of Plant Sciences, Haramaya University, P.O. Box 138, Dire Dawa, Ethiopia; c Melkasa Agricultural Research Center, P.O. Box 436, Adama, Ethiopia

**Keywords:** Bean diseases resistance, *Phaseolus vulgaris*, Severity, Yield and yield components

## Abstract

Common bean is the most important pulse crops worldwide and in Ethiopia for its multipurpose uses. However, common bean production and productivity is mainly constrained by common bacterial blight (CBB) and angular leaf spot (ALS) diseases. Identifying and using resistant common bean genotypes is the best option to reduce the impact of such bacterial and fungal diseases. Field experiments were carried out to evaluate the genetic resistance of 25 common bean genotypes as treatments to CBB and ALS diseases, and agronomic performances at Haramaya and Melkassa, Ethiopia, during the 2019 and 2020 main cropping seasons. The treatments were arranged in a 5 × 5 triple lattice design with three replications. Data on disease intensity, growth, grain yield and yield components were recorded. Analysis of variance indicated significant (p < 0.0001) variations existed among the genotypes for diseases intensity, grain yield and yield components. Eight genotypes, namely DAB-388, DAB-478, DRKDDRB-70, DRKDDRB-81, NUA-225, NUA-517, NUA-536 and NUA-577 attained relatively low disease severity, AUDPC and disease progress rate next to the checks Zoasho (DAB-96) and Gorossa (Biofort large seed-5) to both common bean diseases regardless of locations and seasons. The genotype DAB-525 showed a moderately resistant reaction to both CBB and ALS, and the other genotypes demonstrated variable disease resistance reactions at both locations and in the two cropping years. Similarly, genotypes DAB-388, NUA-225, NUA-517, and NUA-577 relatively executed well for grain yield and yield components at both experimental fields in the 2019 and 2020 main cropping seasons. Disease severity and grain yield variably and negatively associated with reaction groups of genotypes evaluated for reaction CBB and ALS diseases. In the study, resistance to CBB and ALS diseases and good agronomic performing common bean genotypes were identified which could be important for smallholder and commercial bean production in the study area and other similar agro-ecologies in Ethiopia. It is suggested that a large number of common bean accessions should be evaluated in CBB and ALS hot spot agro-ecologies of Ethiopia for more sources of resistance and better agronomic advantages.

## 1 Introduction

Common bean (*Phaseolus vulgaris* L.) is an important food legume comprising both dry and snap beans widely grown in the temperate, tropical and sub-tropical areas of the world (Pamela et al., 2014; Awori et al., 2018). The crop has significant economic importance both in income and food sources with high nutritional value in developing countries of Africa, Asia and Latin America (FAOSTAT, 2020). The crop is rich in protein and micronutrients, such as calcium, folate iron, zinc, magnesium, phosphorus, potassium and vitamin B (Mederos, 2006; Beebe et al., 2014; Petry et al., 2015). In Ethiopia, farmers have been able to adapt, develop and maintain a large genetic diversity to suit their needs in different cropping systems (Shiferaw et al., 2020). The crop is significantly contributing to the national food security and economy. It is consumed in different forms of traditional dishes and in 2014/2015 cropping year, served as source of income in the form of foreign exchange with $100 obtained through exporting of white and red common bean types (Amsalu et al., 2016).

Common bean is produced in Asia (49.5%), Africa (25.7%) and the Americas (24.8%). Globally, Myanmar is the leading producer followed by India and Brazil (FAOSTAT, 2020). In Africa, the highest production is from Ethiopia, Kenya and Uganda (FAOSTAT, 2020). The common bean has diverse growth habit (bush, determinate, indeterminate and climbing type), size (small, medium and large) and color (white, red, sprinkled and others) are grown in the country in which large and small white and red colored beans are the most commonly grown types (Shiferaw et al., 2020). More than 12% of the total grain crop area in Ethiopia was covered with pulse and of which 2.2% was allocated for common bean during the 2019/2020 main cropping season (CSA, 2020).

However, because of several biotic and abiotic factors, the national mean common bean productivity is less than 2 t ha^−1^as compared to the other world mean productivity of 2.5–3.5 t ha^−1^ (CSA, 2020). Among the major biotic production constraints, plant diseases, such as, common bacterial blight [(CBB) (*Xanthomonas axonopodis* pv*. phaseoli* and *X. axonopodis* pv*. phaseoli* var. *fuscans*)], angular leaf spot (*Pseudocercospora griseola*; syn. *Phaeoisariopsis griseola*), anthracnose (*Colletotrichum lindemuthianum*), rust (*Uromyces appendiculatus*), halo blight (*Pseudomonas syringae* pv. *phaseolicola*), and bean common mosaic virus (BCMV) are regarded as economically important and widely distributed in major common bean growing areas of eastern, central and southwestern Ethiopia (Fininsa and Yuen, 2001).

Of the devastating diseases, common bacterial blight and angular leaf spot are commonly occur all the year round and the most important diseases in common bean production areas in Ethiopia (Fininsa and Yuen, 2001; Aytenfisu et al., 2019). These diseases mainly infect the leaves, stems, pods, and common bean seeds. Common bacterial blight is a major seed-borne disease with a range of 30–70% yield losses were recorded on susceptible cultivars worldwide (Karavina et al., 2011). Losses due to CBB are also estimated to range from 10 to 40% on susceptible cultivars in Uganda (Opio et al., 1992), and a 22% yield reduction was reported in sole cropping system in eastern Ethiopia (Fininsa, 2003). On the other hand, ALS produces damage occasionally in USA and Europe, but in Africa, yield losses of 50–60% was reported (Elena et al., 2017). In the absence of ALS disease management measures, yield losses of 45–80% were estimated in Brazil and Colombia under favorable conditions (Guzman et al., 1995). In Uganda, yield losses due to planting ALS susceptible cultivars reached up to 54.7% (Pamela et al., 2014), while more than 47% yield loss was reported due to planting ALS susceptible cultivars in the south and southwestern Ethiopia (Lemessa et al., 2011).

Infected seeds, plant debris, and aerial spread are the primary sources of inocula for infection caused by CBB and ALS diseases (Girma et al., 2022). Thus, the use of the healthy seed, field sanitation, chemicals for spray and seed treatment, soil amendment, proper crop density and crop rotation could be considered and used management options to reduce diseases intensities (Fininsa and Yuen, 2001; Amin et al., 2013). However, crop rotation could be a challenge because of the shortage of cropping land and use of chemicals for spray and seed treatment is often expensive and not readily available to or affordable by smallholder farmers. Therefore, use of genetic resistance is the best strategy, economical and environmentally resilient method of disease management approach in common bean production (Tumsa et al., 2020; Adila et al., 2021). High levels of host resistance could minimize yield losses, reduce use of chemicals, facilitate integrated disease management scheme and increase distribution of pathogen-free seeds (Singh and Schwartz, 2010).

Of course, disease resistance in common bean genotypes is dynamic across environment and overtime. Continuous evaluation and identification of common bean genotypes to two or more diseases renders better protection than relying on a single disease resistance since the occurrence of more than one pathogen on a single crop is a well-known phenomenon in the tropics, and could be used as a primary component in an integrated diseases management scheme (Fininsa and Tefera, 2006). A report by Paparu et al. (2014) indicated that simultaneous infection of common bean by CBB and ALS causal pathogens could result in disease that would exceed 35% severity. In such cases, the effect of a disease complex on yield is estimated that each disease often acts independently and results in a sum up yield losses.

Multiple disease resistance has assumed its importance in recent years because of increased dependence on host plant resistance in integrated disease management systems, and, in addition, because grain legumes, that provide much needed protein, are widely grown in developing countries where access to available resources to farmers are limited. Evaluating common bean genotypes under natural infection for two or more diseases to identify elite genotypes or sources of resistance that can be used for production or regularly introgressed into commercial cultivars are important (Fininsa and Tefera, 2006). This could counteract the newly emerging plant pathogenic races and could reinforce resistance in the already existing resistant cultivars.

Identifying the disease resistance status of elite common bean genotypes, which have promising high yield and yield components, is imperative to support increased common bean production and productivity (Adila et al., 2021). Farmers prefer quality genotypes possessing large seeds with good taste and fast cooking characteristics in Ethiopia. Previously breeding attempts were made only for one genetic resistance in common bean genotype improvement; hence, research information on the genetic resistance of the crop for two or more diseases is overlooked. Therefore, periodic evaluations of common bean genotypes in different agro-ecologies, where different diseases prevail are inevitable for identifying economically important traits. Thus, the specific objective of this study was to evaluate selected common bean genotypes for their resistance to CBB and ALS diseases under natural infection and agronomic performances.

## 2 Materials and methods

### 2.1 Description of the experimental sites

Field experiments were carried out at Haramaya University crop research station (Raare) and Melkassa Agricultural Research Center (MARC, Melkassa), Ethiopia, during the 2019 and 2020 main cropping seasons. Haramaya University is located at 9°26′N latitude and 42°30′E longitude at an altitude of 2006 m above sea level in eastern Ethiopia. The site is one of the major common bean production areas in the region. It receives a mean annual rainfall of 790 mm with minimum 16 °C and maximum 24.5 °C mean annual temperatures, respectively. Alluvial is a dominant soil type with 3.1% OM and 7.7 pH (Fininsa and Tefera, 2006). Melkassa is found in the semi-arid region of the central Rift Valley, another major common bean production area in Ethiopia. It is located at 8°24′N latitude, 39°12′E longitude, and at an altitude of 1550 m above sea level. The site receives an average of 915.7 mm annual rainfall and the minimum and maximum annual mean temperatures of 13.8 and 28.9 °C, respectively. The soil type of the site is Andosols (Moges et al., 2019).

### 2.2 Weather data

**Figure 1:**
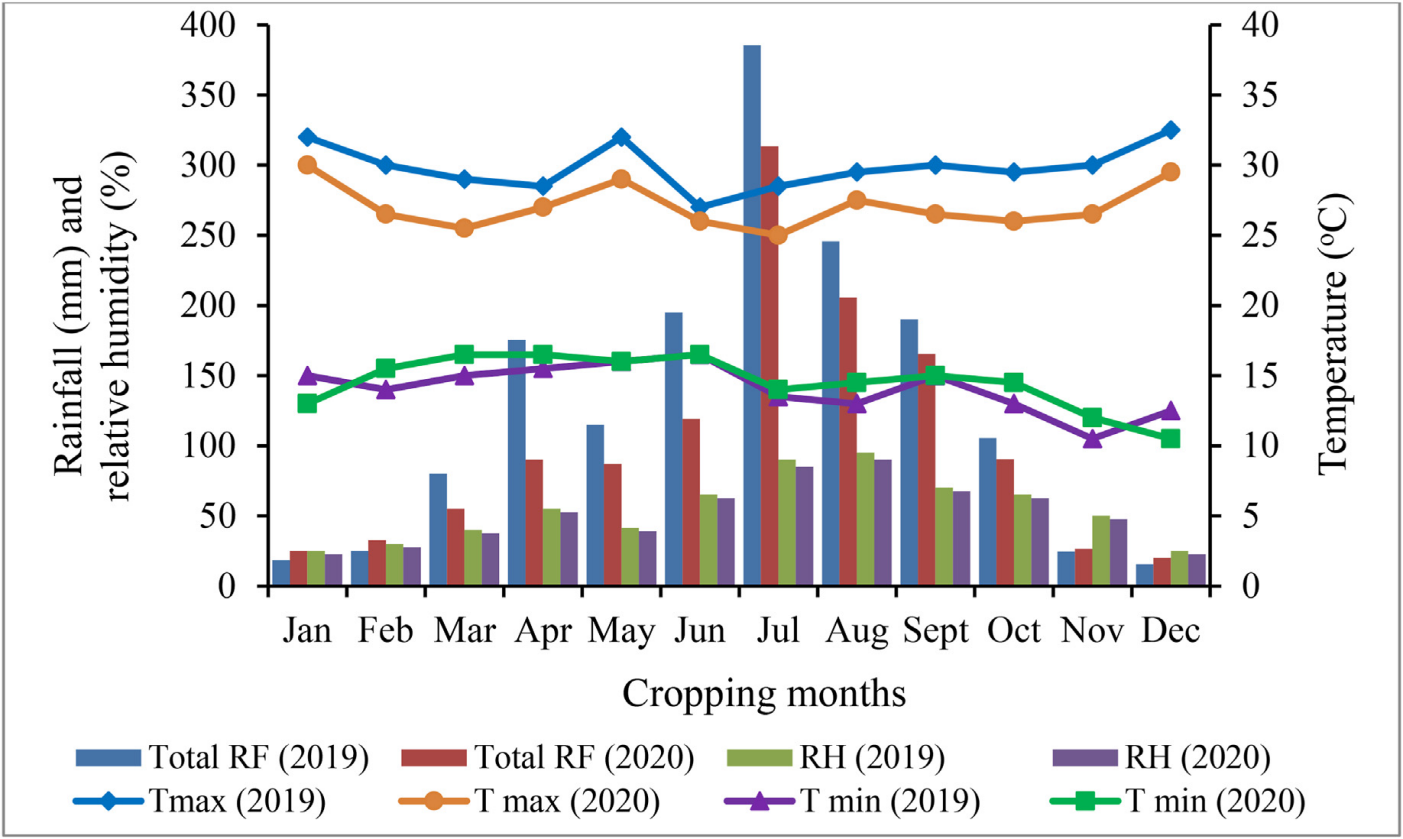
Monthly total rainfall (mm), maximum and minimum temperatures (^o^C) and relative humidity (%) of Haramaya, Ethiopia during the 2019 and 2020 main cropping seasons.

**Figure 2:**
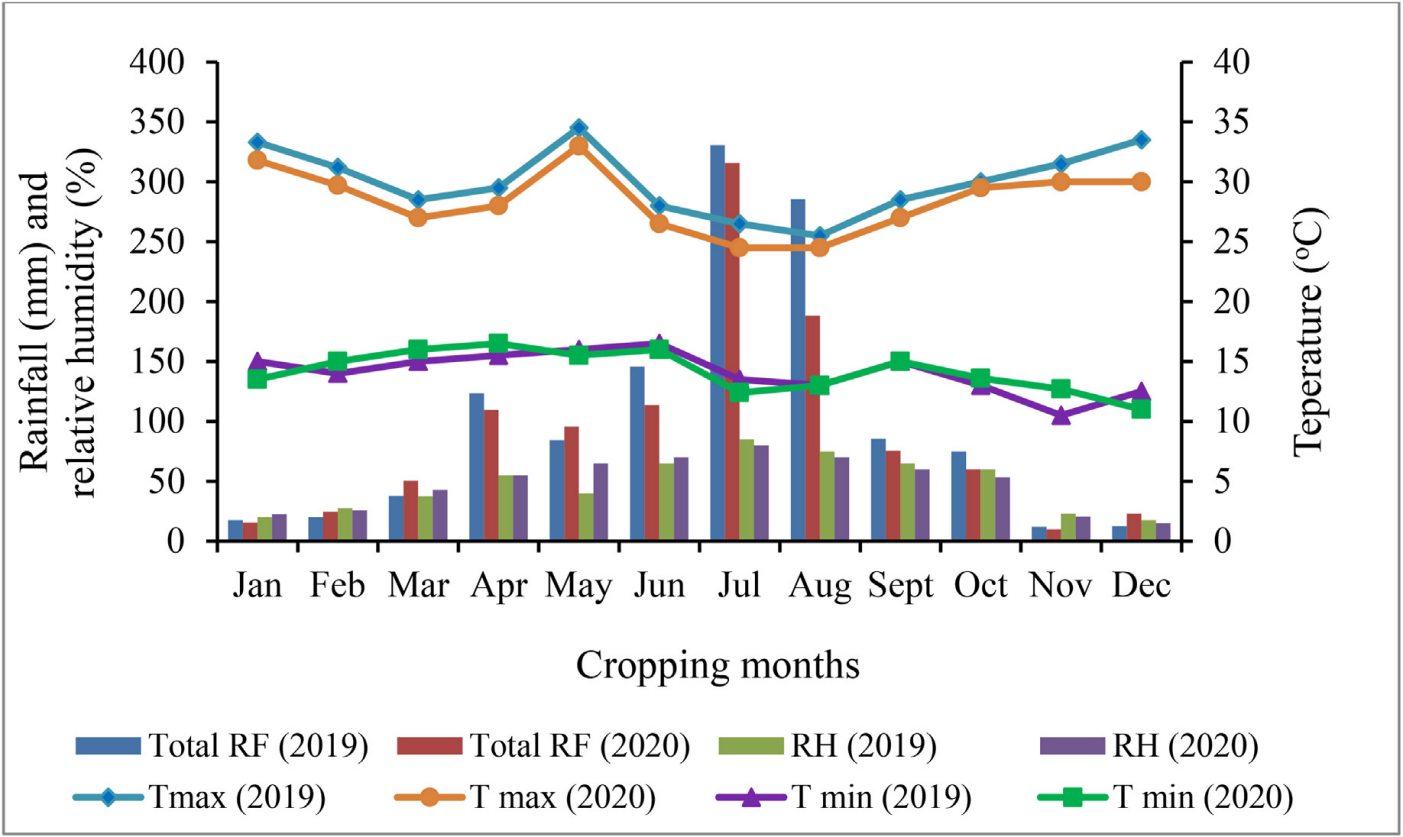
Monthly total rainfall (mm), maximum and minimum temperatures (^o^C) and relative humidity (%) of Melkassa, Ethiopia during the 2019 and 2020 main cropping seasons.

Monthly minimum and maximum temperatures (^o^C), rainfall (mm) and relative humidity (%) of the 2019 and 2020 cropping seasons of Haramaya and Melkassa data were obtained from the nearby meteorological stations and are illustrated hereunder in Figures 1 and 2. During the growing periods, Haramaya received a total rainfall of 231.6 mm in 2019 and 193.7 mm in 2020 (Figure 1). Similarly, Melkassa area received a total rainfall of 194.2 and 159.8 mm in 2019 and 2020, respectively (Figure 2).

### 2.3 Treatments, experimental design, and field management

A total of 23 white and red colored large seeded common bean genotypes obtained from Melkassa Agricultural Research Center, namely, DRKDDRB-65, DRKDDRB-70, DRKDDRB-81, CCSS6915-11-32, CCSS6915-11-38,DAB-245,DAB-278,DAB-283,DAB-366, DAB-379, DAB-388, DAB-478,DAB-525, DAB-545,NUA-225,NUA-353, NUA-517, NUA-536,NUA-577, RNSS 6915-89-26, RNSS6915-89-33, SAB-632 and SAB-736, and two checks Gorossa (Biofort large-seeded-5) and Zoasho (DAB-96) were evaluated for their reaction to multiple diseases and agronomic performances under natural rain-fed conditions at the two testing locations. The checks were released by MARC in 2017 for their disease (common bacterial blight, angular leaf spot, and halo blight) resistance and good grain yield performances. All the 23 genotypes used have determinate growth habit and were introduced to MARC from the International Center for Tropical Agriculture (CIAT) for various screening studies.

At each experimental site, bean fields regularly followed potato and maize crops at Haramaya and Melkassa, respectively. Genotypes were planted in four rows per plot and 20 plants were maintained per row. The plot size was 1.6 m × 2 m (3.2 m^2^) where inter-row spacing was 0.4 m and intra-row spacing was 0.1 m. The planting date was on 10 July 2019 and 14 July 2020 at Haramaya; and on 6 July 2019 and 11 July 2020 at Melkassa. Sowing was done by placing two seeds per hill and then, thinning to one plant per hill to obtain recommended plant density. The treatments were arranged in a 5 × 5 triple lattice design with three replications. Inorganic blended fertilizer (NPS) was applied at a rate of 100 kg ha^−1^ during planting. Other agronomic practices were treated as non-experimental variables and as per national recommendation for common bean.

### 2.4 Common bean bacterial blight and angular leaf spot assessment

Common bean bacterial blight and ALS disease severities were recorded and the area under disease progress curves (AUDPC) and disease progress rates were separately computed for Haramaya and Melkassa, for the 2019 and 2020 main cropping seasons. Disease severity data for both CBB and ALS disease were recorded from 10 randomly selected plants in the central rows of each plot. Disease severity was recorded five times seven days interval beginning from the first onset of disease symptoms. Common bean bacterial blight and ALS disease severities were scored based on a 1–9 disease scale; where, 1 = 0% of the leaves or pods infected, 2 = 2%, 3 = 5%, 4 = 10%, 5 = 20%, 6 = 25%, 7 = 50%, 8 = 75%, and 9 = 85% (CIAT, 1987). Symptoms, which appeared as small water-soaked spots bordered by chlorotic zones on leaves, and circular and brownish red spots on pods were considered for CBB assessment. The assessment was begun 49 days after planting (DAP) at Haramaya and 46 DAP at Melkassa in the 2019 main cropping season. In the 2020 main cropping season, CBB severity assessment was begun 52 DAP at Haramaya and 48 DAP Melkassa. Following brown spots on leaves and reddish-brown to black circular spots on pods of common bean genotypes, ALS assessment was started at Haramaya at 47 and 51 DAP in the 2019 and 2020 main copping season, respectively. At Melkassa, ALS assessment was begun on 44 DAP in 2019 and on 49 DAP in 2020 main cropping season. The severity scores were converted into percentage severity index (PSI) using the formula suggested by Wheeler (1969).

$$\text{PSI}=\frac{\text{Sum ​of ​numerical ​ratings}}{\text{Total ​number ​of ​plants ​rated}\times \text{maximum ​score ​on ​the ​scale}}\times 100$$

The resistance reaction type categories were determined from mean values of disease severity scores for each genotype per plot. In addition, severity scores at R8 (plants began to fill seeds in their pods or pod filling growth stage) were also used for classifying the genotypes into different reaction categories as resistant (R), moderately resistant (MR) and susceptible (S) as suggested by van Schoonhoven and Pastor-Corrales (1987) and CIAT (1987). Mean severity scores of ≤3, 4–6 and ≥7 were considered as R, MR and S, respectively (CIAT, 1987). The PSI values were used for analysis in the area under disease progress curve (AUDPC) that was calculated for each plot using the formula of Campbell and Madden (1990). Values of AUDPC were used in the analysis of variance to compare the amount of disease and associated pressure among genotypes during the epidemic periods.

$$\text{AUDPC}=\overset{\text{n}-1}{\underset{\text{i}=1}{\sum }}\left(\frac{x_{\text{i}}+x_{\text{i}+1}}{2}\right)\left(t_{\text{i}+1}-t_{\text{i}}\right)$$

Where, *x*_i_ = disease severity (%) recorded at the i^th^ observation, t_i_ = time of the i^th^ assessment in days and n = total number of observations. Thus, AUDPC was expressed in %-days, since severity was expressed in percent and time in days.

### 2.5 Common bean growth assessment

Crop phenology, such as, days to 50% emergence, days to 50% flowering, days to 90% physiological maturity and plant height was recorded from the two central rows of each plot. Days to 50% emergence was determined by counting the number of days to 50% emergence of sown seeds, and days to 50% flowering were determined by counting the number of plants to 50% of grown plants flowered per plot. Days to maturity were recorded by counting the number of days taken from emergence to the days that 90% of the plants in the plot were physiologically matured. Plant height (cm) was measured using a meter at the physiological maturity stage from the harvestable rows of 10 randomly taken plants. Stand count at harvest was also recorded by counting the number of plants present per plot at harvest. Mean values of each growth parameter were used for statistical analysis.

### 2.6 Grain yield and yield components assessment

Yield components, including the number of pods per plant (NPPP) and the number of seeds pod (NSPP), were collected from the central two rows of each plot. The number of pods per plant was recorded by counting the number of pods from 10 randomly taken plants per plot. Numbers of seeds per pod were recorded by counting the number of seeds from pods of 10 plants per plot. Pods were threshed and the total grain weight obtained from each plot was adjusted to 12% moisture content and converted into tons per hectare (ISTA, 1996). Hundred seed weight (HSW) was determined from composite samples taken from each plot of the harvested total grain yield and determined by sensitive balance after adjusting to 12% moisture content (ISTA, 1996).

### 2.7 Data analyses

Disease severity, AUDPC, common bean growth, yield and yield components data were subjected to analysis of variance (ANOVA) using SAS GLM procedure version 9.2 (SAS, 2014). Mean separation among genotypes was performed using Duncan's Multiple Range Test (DMRT) at 5% probability level (Gomez and Gomez, 1984). Logistic [ln (y/(1–y))] (Van der Plank, 1963) and Gompertz [–ln (–ln(y))] (Berger, 1981) models were evaluated to determine the disease progress rates from the linear regression of disease severities versus days after planting (DAP) for each genotype. The fitness of the models was tested based on the magnitude of the coefficient of determination (R^2^) and standard error (SE) (Campbell and Madden, 1990). The logistic model was better fitted with higher R^2^ and lower SE data than the Gompertz model at both locations in both years. Hence, CBB and ALS progress rates were estimated and compared using the logistic model. The two locations and seasons were considered as different environments because of the heterogeneity of variances as tested using Bartlett's test and the F-test was significant for the parameters analyzed (Gomez and Gomez, 1984). Thus, data were analyzed separately.

## 3 Results

Combined analyses of disease parameters, growth, grain yield and yield components data were showed significant variation between experimental locations and cropping seasons except for days to 50% emergence and stand count at harvest. Therefore, results were separately presented for disease, growth, yield and yield components for each location and cropping season (Table 1).

**Table 1:** Combined analyses of mean squares and significant tests of PSI and AUDPC for common bean bacterial blight (*X. axonopodis* pv. *phaseoli*), angular leaf spot (*P. griseola*), growth, yield and yield components of common bean genotypes at Haramaya and Melkassa, Ethiopia, during the 2019 and 2020 main cropping seasons.

Sources of variation^a^	df	Disease parameters^b^				Growth, yield and yield components^c^							
		CBB		ALS									
		PSI	AUDPC	PSI	AUDPC	DF	PH (cm)	DM	NSPP	NPPP	SCH	HSW (g)	GY (t ha^−1^)
Year	1	3576.2∗∗	1707792.6^ns^	12656.8^ns^	751220.4∗∗	19.3∗	66.2∗	167.5∗∗	10.2∗∗	273.8∗	8.0^ns^	735.1∗∗	20.9∗∗
Rep	2	235.7∗	36337.9^ns^	1053.8∗∗	315919.3∗∗	19.4^ns^	121.7∗∗	63.8∗	3.5∗	22.9∗	11.1^ns^	640.6∗∗	0.2 ^ns^
Block (r)	12	482.5∗∗	60223.8∗∗	483.1∗∗	60919.4∗∗	55.6∗∗	112.3∗∗	15.1∗	6.1∗∗	26.6∗∗	76.7^ns^	467.3∗∗	2.8∗∗
Location	1	1152.4∗∗	166348.7∗∗	2392.2∗∗	6727.0∗	10.4∗	1787.5∗	117.8∗	12.7∗	240.1∗	14.6^ns^	510.6∗∗	2.92∗∗
Genotype	24	118.5∗∗	15607.7∗∗	266.8∗	15233.9∗	22.1∗∗	36.8∗∗	8.9∗	2.6∗∗	28.7∗	26.4^ns^	268.9∗	1.5∗∗
Y x G	24	123.1∗	7925.2∗	152.3∗	11264.1∗	1.7∗	78.6∗	24.6∗	12.2∗	17.5∗	4.1^ns^	119.5∗	5.2∗
L x G	24	49.3∗	8867.7∗	232.5∗	8487.4∗	2.4∗	95.7∗	13.8∗	1.9∗	20.3∗	15.9^ns^	161.2∗	2.6∗
Y x L x G	24	89.1∗	10816.2∗	856.1∗∗	10174.3∗	2.6∗	120.4∗	7.1∗	13.2∗	16.2∗	15.9^ns^	321.8∗	6.4∗
Error	186	48.8	5321.1	76.2	6675.4	6.5	14.6	4.7	1.1	8.9	11.09	55.9	0.4
Mean		37.94	363.6	37.6	342.3	47.5	42.4	88.9	5.0	12.8	28.83	49.6	3.4
CV (%)		18.4	20.1	23.2	17.1	5.3	9.1	22.3	20.1	23.2	11.54	15.1	19.1
R^2^ (%)		71.9	77.3	78.4	81.7	70.3	76.7	82.3	71.2	72.6	54.6	74.1	80.1

a Rep = Replication; Y = Year, G = Genotypes (treatment); CV = Coefficient of variation; L = Location; and R^2^ = Coefficient of determination.

b CBB = Common bean bacterial blight; PSI = Percent severity index; ALS = Angular leaf spot; and AUDPC = Area under disease progress curve.

c DF = Days to 50% flowering; PH = Plant height; DM = Days to 90% physiological maturity; NSPP = Number of seeds per pod; NPPP = Number of pods per plant; SCH = Stand count at harvest; HSW = Hundred seed weight; and GY = Grain yield; df = degrees of freedom; ns = not significant; and∗ and ∗∗ significant at p < 0.05 and p < 0.01, respectively.

### 3.1 Common bean bacterial blight severity

The genotypes DAB-283, DAB-525, RNSS6915-89-26 and RNSS6915-89-33, showed small water-soaked spots bordered by chlorotic zones on leaves and circular and brownish red spots on pods at 49 DAP at Haramaya and 46 DAP at Melkassa in 2019. In 2020, the first CBB severity recording was started at 52 DAP at Haramaya and 48 DAP at Melkassa. None of the evaluated common bean genotypes were immune to CBB, irrespective of variable responses among the genotypes across locations and over cropping seasons.

The analysis of variance revealed that genotypes maintained highly significant (p < 0.0001) differences for final disease severity at both locations in the two cropping seasons (Table 2). At Haramaya, the mean final CBB severity ranged from 22.75% to 48.56% in 2019 and from 20.22% to 44.21% in 2020. The highest (48.56%) CBB severity was recorded on genotype RNSS6915-89-26, followed by RNSS6915-89-33 (46.71%), DAB-283 (44.48%) and DAB-525 (43.43%) during the 2019 cropping season. In 2020, the highest final CBB severity index was reduced by 8.95%, 11.11%, 7.3% and 16.73% compared to 2019 the highest final CBB severity of respective genotypes. Few spots surrounded by necrosis were recorded on leaves of both check Zoasho (DAB-96) which is reduced by 53.15%, followed by check Gorossa (Biofort large seed-5) by 50.56%, NUA-225 (46.62%) and NUA-517 (44.41%) lower final severity in the 2019 cropping season respective genotypes. During the 2020 cropping season, the lowest final CBB severity was recorded on the 2019 recorded genotypes, which was reduced by 15.34%, 4.34%, 5.43%, and 5.38%. A similar trend was seen at Melkassa during the two cropping seasons for CBB final mean severity.

**Table 2:** Mean PSI and AUDPC (%-days) of common bean bacterial blight (*X. axonopodis* pv. *phaseoli*) on common bean genotypes at Haramaya and Melkassa, Ethiopia, during the 2019 and 2020 main cropping seasons.

Genotype	Disease parameters at Haramaya^1^				Disease parameters at Melkassa^1^				
	PSI		AUDPC (%-days)		PSI		AUDPC (%-days)		
	2019	2020	2019	2020	2019	2020	2019	2020	
CCSS6915-11-32	29.68^de^	22.53^h–j^	282.18^j^	265.86^i–l^	24.81^f–i^	22.22^g–j^	247.85^mn^	268.67^j–l^	
CCSS6915-11-38	28.18^d–g^	23.15^g–j^	275.36^jk^	280.30^g–i^	25.67^e–h^	23.22^f–i^	267.68^hi^	271.92^j–l^	
DAB-245	34.46^c^	30.14^c^	261.78^mn^	307.27^de^	32.22^b^	26.67^d^	287.75^d^	351.16^e^	
DAB-278	29.68^de^	27.93^cd^	332.07^d^	318.60^d^	30.37^c^	26.44^d^	283.89^d–f^	369.57^d^	
DAB-283	44.48^b^	41.23^a^	433.42^bc^	419.17^b^	35.56^b^	35.45^a^	429.42^b^	404.98^b^	
DAB-366	29.71^de^	22.72^f–i^	299.10^f–h^	257.6^j-m^	26.47^d–f^	25.62^d–f^	272.96^gh^	305.57^f^	
DAB-379	27.44^d–h^	21.5^h–j^	292.17^hi^	267.66^i–k^	31.11^c^	23.42^e–h^	291.52^de^	265.89^j–l^	
DAB-388	28.69^c–e^	24.61^e–j^	267.48^lm^	294.54^e–g^	28.84^c–e^	25.80^d–f^	279.40^e–g^	350.53^e^	
DAB-478	28.31^d–g^	25.84^h–j^	264.58^mn^	247.96^l–n^	25.93^d–g^	23.53^e–h^	261.66^i–k^	297.02^f–h^	
DAB-525	43.43^b^	36.16^b^	431.77^c^	397.41^c^	35.56^b^	36.49^a^	393.88^c^	307.18^c^	
DAB-545	28.56^d–f^	26.19^c–e^	303.61^f^	275.34^h-k^	24.44^f-i^	21.97^h–j^	254.56^j–l^	269.38^j–l^	
DRKDDRB-65	28.87^d–f^	22.47^h–j^	301.76^fg^	288.10^f–h^	27.12^d–f^	22.62^g–i^	265.51^h–j^	312.6^f^	
DRKDDRB-70	25.21^f–j^	22.24^h–j^	274.45^kl^	262.56^i-l^	25.75^e–h^	25.80^de^	275.5^f–h^	304.78^f^	
DRKDDRB-81	27.69^d–h^	26.58^d–f^	248.45^p^	256.63^k–m^	23.33^g–i^	22.22^g–j^	274.06^f–h^	281.27^h–k^	
NUA-225	23.74^h–j^	21.17^h–j^	253.4^op^	238.75^m–o^	22.22^i^	20.0^j–l^	244.18^no^	264.57^kl^	
NUA-353	27.44^d–h^	24.98^d–g^	291.11^i^	279.70^g–i^	24.78^f–i^	24.78^d–g^	260.24^i–k^	285.12^g–i^	
NUA-517	24.14^g–j^	21.46^h–j^	257.48^no^	241.76^m–o^	22.45^i^	20.74^i–k^	250.38^l–n^	268.33^j–l^	
NUA-536	26.96^e–i^	23.39^e–h^	297.62^f–i^	276.6^g-j^	26.66^d–f^	22.36^g–j^	256.45^j–m^	301.33^fg^	
NUA-577	24.81^f–j^	21.53^h–j^	294.23^g–i^	248.26^lmn^	22.84^hi^	20.82^i–k^	291.97^d^	276.99^i–k^	
RNSS 6915-89-26	48.56^a^	44.21^a^	484.64^a^	473.12^a^	40.46^a^	38.38^a^	467.95^a^	455.68^a^	
RNSS 6915-89-33	46.71^ab^	41.52^a^	439.34^b^	427.51^b^	37.97^a^	37.04^bc^	449.62^a^	438.33^b^	
SAB-632	28.91^d–f^	27.26^c–e^	294.03^g–i^	312.03^de^	24.44^f–i^	24.37^d–h^	270.87^g–i^	283.78^g–k^	
SAB-736	31.51^cd^	27.57^cd^	311.21^e^	301.91^d–f^	28.65^c–e^	23.48^e–h^	290.27^d^	294.5^f–i^	
Gorossa (Biofort L-5)	23.23^ij^	20.76^j^	241.01^q^	234.41^no^	22.22^i^	19.51^kl^	234.76^op^	256.32^l^	
Zoasho (DAB-96)	22.75^j^	20.22^j^	238.63^q^	224.9^o^	19.26^j^	17.78^l^	228.26^p^	235.25^m^	
CV (%)	3.45	6.85	4.24	3.45	5.84	5.22	2.05	3.27	
R^2^ (%)	98.8	97.2	99.8	98.7	95.2	97.6	99.5	98.6	
P-value	<0.0001	<0.0001	<0.0001	<0.0001	<0.0001	<0.0001	<0.0001	<0.0001	
Eff. to RCBD (%)	90.51	94.5	92.1	95.4	91.3	84.18	93.7	97.7	

1 Final PSI were recorded at 77 DAP (2019) and 80 DAP (2020) at Haramaya and 74 DAP (2019) and 76 DAP (2020) at Melkassa; and AUDPC = Area under disease progress curve; CV = Coefficient of variation; R^2^ = Coefficients of determination; and Eff. to RCBD = Efficiency of lattice relative to randomized complete block design. Means followed by the same letter(s) are not significantly different at p < 0.05.

At Melkassa, the final mean CBB severity ranged from 19.26 to 40.46% in 2019 cropping season and from 17.78 to 38.38% in 2020 cropping season. The highest (38.38%) mean CBB severity was recorded on the genotype RNSS6915-89-26, while the lowest was recorded from check Zoasho (DAB-96) (19.26%) and on Gorossa (Biofort large-seeded-5), which resulted in 22.22% final disease severity (Table 2). Zoasho (DAB-96) and Gorossa (Biofort large-seeded-5) reduced disease severity by 56.05 and 45.08%, respectively, as compared to the genotype RNSS6915-89-26. The same trend was noticed regarding disease severity at Melkassa in the 2020 cropping season (Table 2).

### 3.2 Common bacterial blight AUDPC

The analysis of variance indicated that there were significant (p < 0.001) differences among genotypes, locations and the cropping seasons for AUDPC values (Table 2). The highest (484.64%-days) AUDPC value was computed from the genotype RNSS6915-89-26, followed by RNSS 6915-89-33 (439.34%-days) at Haramaya in 2019 main cropping season. In the same cropping season, planting NUA-225 and NUA-517 lowered AUDPC by 47.71%-days and 46.87%-days, respectively, next to the checks Zoasho (DAB-96) (50.76%-days) and Gorossa (Biofort large-seeded-5) (50.27%-days) as compared to RNSS6915-89-26 at Haramaya. Similarly, AUDPC values reduced by 49.53% (NUA-225) and 48.9% (NUA-517) next to the checks Zoasho (DAB-96) (52.46%-days) and Gorossa (Biofort large-seeded-5) (50.45%-days) compared to genotype RNSS6915-89-26 at Haramaya in the 2020 cropping season (Table 2). At Melkassa, the highest (467.95%-days) AUDPC value was also calculated for the genotype RNSS6915-89-26, followed by RNSS 6915-89-33 (449.62%-days), while the lowest AUDPC values were recorded on check genotypes Zoasho (DAB-96) (228.26%-days) and Gorossa (Biofort large-seeded-5) (234.76%-days) in 2019 cropping season. In 2020, the highest AUDPC values were computed for the same genotypes with lower AUDPC values than in the 2019 cropping season (Table 2).

### 3.3 Common bean bacterial blight progression rate (r)

Disease progress rates and parameter estimates of CBB at Haramaya and Melkassa during both 2019 and 200 main cropping seasons are presented hereunder Table 3. The disease progression showed variation among the genotypes evaluated for reaction to CBB at both locations in the two seasons. Disease progress rates ranged from 0.009 to 0.041 units day^−1^ at Haramaya and from 0.008 to 0.040 units day^−1^at Melkassa were computed for the 2019 main cropping season. The genotypes had disease progress rates lay between 0.007 and 0.0.038 units day^−1^ at Haramaya and 0.006 and 0.035 units day^−1^at Melkassa in the 2020 main cropping season. The genotype RNSS6915-89-26 demonstrated the fastest CBB disease progress rate to reach the highest disease pressure compared with other genotypes regardless of locations and seasons.

**Table 3:** Disease progress rate (units day^−1^) of common bean bacterial blight (*X. axonopodis* pv. *phaseoli*) on common bean genotypes at Haramaya and Melkassa, Ethiopia, during the 2019 and 2020 main cropping seasons.

Genotype	Progression of CBB at Haramaya^1^						Progression of CBB at Melkassa^1^					
	2019			2020			2019			2020		
	DPR	SE of rate	R^2^ (%)	DPR	SE of rate	R^2^ (%)	DPR	SE of rate	R^2^ (%)	DPR	SE of rate	R^2^ (%)
CCSS6915-11-32	0.022	0.029	99.3	0.020	0.030	94.7	0.030	0.059	97.5	0.023	0.018	97.7
CCSS6915-11-38	0.024	0.034	98.9	0.023	0.029	93.4	0.024	0.044	98.8	0.022	0.016	96.5
DAB-245	0.020	0.042	97.4	0.024	0.035	88.9	0.030	0.062	98.8	0.022	0.021	95.5
DAB-278	0.019	0.019	94.6	0.018	0.029	92.4	0.026	0.058	94.8	0.023	0.015	91.9
DAB-283	0.033	0.030	92.4	0.030	0.036	90.0	0.035	0.053	98.7	0.030	0.017	94.6
DAB-366	0.025	0.032	97.8	0.018	0.027	94.1	0.029	0.056	98.7	0.015	0.018	97.5
DAB-379	0.023	0.029	90.5	0.026	0.025	97.7	0.031	0.060	99.1	0.019	0.018	99.5
DAB-388	0.026	0.030	99.2	0.019	0.026	96.8	0.019	0.063	96.7	0.019	0.019	95.7
DAB-478	0.013	0.042	98.4	0.017	0.035	92.5	0.018	0.072	98.9	0.016	0.023	96.3
DAB-525	0.031	0.017	98.8	0.029	0.032	94.4	0.036	0.064	92.7	0.032	0.016	95.3
DAB-545	0.015	0.014	99.1	0.016	0.032	96.2	0.016	0.063	93.6	0.016	0.015	92.5
DRKDDRB-65	0.024	0.031	99.3	0.024	0.026	90.0	0.030	0.053	91.4	0.022	0.017	96.6
DRKDDRB-70	0.024	0.035	92.8	0.020	0.029	91.1	0.017	0.059	94.6	0.024	0.019	95.5
DRKDDRB-81	0.014	0.030	96.7	0.014	0.026	92.6	0.016	0.053	94.5	0.021	0.017	98.2
NUA-225	0.012	0.029	97.4	0.013	0.025	94.4	0.010	0.051	94.2	0.014	0.016	96.4
NUA-353	0.026	0.042	95.7	0.031	0.035	88.6	0.025	0.024	90.0	0.021	0.013	98.3
NUA-517	0.013	0.037	96.6	0.015	0.031	96.6	0.014	0.064	97.8	0.019	0.020	98.3
NUA-536	0.022	0.033	92.4	0.025	0.029	89.7	0.024	0.011	98.8	0.026	0.009	94.6
NUA-577	0.015	0.032	98.1	0.020	0.027	92.3	0.015	0.056	93.2	0.020	0.018	93.6
RNSS 6915-89-26	0.041	0.038	98.7	0.038	0.029	87.8	0.040	0.070	94.6	0.035	0.022	93.5
RNSS6915-89-33	0.037	0.041	97.6	0.034	0.034	89.6	0.038	0.066	96.6	0.034	0.021	96.7
SAB-632	0.021	0.028	95.1	0.022	0.023	88.9	0.028	0.048	98.9	0.021	0.015	98.1
SAB-736	0.020	0.022	99.8	0.022	0.014	87.8	0.027	0.049	96.7	0.023	0.014	96.7
Gorossa (Biofort L-5)	0.010	0.027	96.6	0.010	0.018	91.3	0.009	0.029	98.8	0.011	0.011	96.6
Zoasho (DAB-96)	0.009	0.015	97.9	0.007	0.020	96.6	0.008	0.019	91.9	0.006	0.007	97.8

1 CBB = Common bacterial blight; DPR = Disease progress rate, and SE = Standard error of rate; and R^2^ = Coefficient of determination.

Generally, the CBB progression rates were relatively higher at Haramaya than at Melkassa in both cropping seasons (Table 3). The fastest disease progression rate on the genotype RNSS6915-89-26 at Melkassa was 0.024 units day^−1^, which was slower than the rate calculated for the same genotype at Haramaya in 2019 main cropping season. Similarly, the same genotype exhibited CBB progression rate of 0.079 units day^−1^ which was once again much decreased at Melkassa, compared to the rate calculated at Haramaya in 2020 main cropping season. For example, the CBB progression rate on genotype Zoasho (DAB-96) at Melkassa in 2019 main cropping season was 0.22 units day^−1^ slower than at Haramaya in the same 2019 cropping season. Similarly, the CBB progression rate 0.25 units day^−1^on the genotype Zoasho (DAB-96) was higher at Haramaya in 2020 cropping season than the CBB progression rate at Melkassa in the same 2020 cropping season.

### 3.4 Angular leaf spot severity

The first ALS symptoms, small brown spots and reddish-brown to black circular spots on leaves, were observed on the genotypes DAB-245, DAB-379 and DAB-525 at 47 DAP at Haramaya in 2019 main cropping season and 51 DAP in 2020 cropping season. At Melkassa, the first ALS symptoms were also observed on the same genotypes at 44 DAP in 2019 main cropping season and at 49 DAP in 2020 cropping season. Once again, none of the common bean genotypes was immune to ALS despite the genotypic variations for ALS pressure was seen at both locations and cropping seasons. The analysis of variance indicated that the genotypes studied established a highly significant (p < 0.0001) genotypic variability for ALS final severity at both locations during the two seasons (Table 4).

**Table 4:** Mean PSI and AUDPC (%-days) of angular leaf spot (*P. griseola*) on common bean genotypes at Haramaya and Melkassa, Ethiopia, during the 2019 and 2020 main cropping seasons.

Genotype	Disease parameters at Haramaya^1^				Disease parameters at Melkassa^1^			
	PSI		AUDPC (%-days)		PSI		AUDPC (%-days)	
	2019	2020	2019	2020	2019	2020	2019	2020
CCSS6915-11-32	24.63^h–j^	22.94^fg^	266.17^jk^	288.63^f–h^	22.95^h–i^	25.66^fg^	291.57^h^	265.59^k^
CCSS6915-11-38	29.63^de^	23.03^fg^	263.18^lm^	295.88^e–g^	25.27^e–h^	28.89^cd^	304.18^fg^	288.71^g–j^
DAB-245	40.24^a^	37.51^a^	427.86^a^	412.89^a^	38.38^a^	35.56^a^	418.71^a^	413.02^a^
DAB-278	23.63^i–k^	24.78^d–f^	276.39^hi^	277.11^ij^	25.56^d–h^	28.89^cd^	313.98^d–f^	283.55^h–k^
DAB-283	31.11^b–d^	25.98^cd^	307.97^cd^	313.98^d^	30.86^c^	29.39^cd^	321.91^d^	313.33^de^
DAB-366	26.44^f–h^	25.34^de^	301.22^e^	281.64^h–j^	27.25^b–e^	27.83^de^	278.37^ij^	327.33^d^
DAB-379	33.33^b^	28.19^b^	331.84^c^	311.24^c^	34.34^b^	30.14^b^	355.12^b^	361.86^c^
DAB-388	27.54^e–g^	25.91^cd^	269.16^jk^	265.92^kl^	24.56^f–i^	23.17^h^	303.12^g^	301.73^e–h^
DAB-478	23.13^i–k^	20.04^hi^	249.98^n^	257.86^lm^	20.91^h^	20.96^jk^	261.73^k^	247.13^l^
DAB-525	38.38^a^	35.56^a^	407.81^b^	392.06^b^	36.89^a^	32.22^a^	409.94^a^	389.65^b^
DAB-545	26.19^f–h^	21.56^g–i^	258.23^m^	273.84^jk^	23.35^g–j^	25.45^fg^	281.18^i^	274.6^jk^
DRKDDRB-65	24.44^h–k^	23.21^e–g^	274.02^ij^	275.59^i–k^	26.67^c–f^	30.12^c^	355.29^b^	276.46^i–k^
DRKDDRB-70	25.18^g–j^	25.15^d–f^	270.64^jk^	276.06^i–k^	24.44^f–i^	25.52^fg^	305.73^fg^	310.22^d–f^
DRKDDRB-81	21.88^k^	18.52^i^	246.77^n^	255.19^m^	19.65^h^	20.49^k^	245.56^l^	238.49^l^
NUA-225	25.59^f–j^	21.48^g–i^	276.74^hi^	278.82^h–j^	25.4^d–h^	26.67^ef^	314.77^d–f^	300.50^e–h^
NUA-353	27.54^e–g^	22.96^fg^	281.53^gh^	298.01^ef^	28.53^bc^	22.22^i–k^	351.33^b^	294.87^e–i^
NUA-517	24.96^g–j^	21.25^g–i^	298.98^e^	284.30^h–j^	23.68^g–j^	25.21^fg^	308.47^e–g^	290.7^f–j^
NUA-536	25.95^f–i^	22.22^gh^	259.96^m^	279.92^h–j^	25.56^d–h^	24.68^f–h^	299.41^gh^	299.89^e–h^
NUA-577	26.67^f–h^	25.23^d–f^	266.6^kl^	283.43^h–j^	25.52^d–h^	27.67^de^	317.52^de^	303.72^e–g^
RNSS 6915-89-26	32.22^bc^	27.65^bc^	303.65^de^	320.32^d^	30.87^c^	29.61^cd^	334.26^c^	326.02^d^
RNSS 6915-89-33	30.87^d^	25.51^c–e^	298.77^e^	310.45^d^	30.14^b^	27.67^b–e^	321.88^d^	313.0^de^
SAB-632	27.86^ef^	22.98^fg^	282.58^g^	299.94^e^	25.92^c–g^	26.67^ef^	316.43^de^	312.59^de^
SAB-736	24.63^h–j^	25.51^c–e^	288.93^f^	286.64^g–i^	28.34^b–d^	24.44^gh^	300.99^gh^	308.98^d–f^
Gorossa (Biofort L-5)	24.31^h–k^	20.95^g–i^	263.03^lm^	260.74^lm^	21.46^jk^	23.93^gh^	271.22^i–k^	279.17^i–k^
Zoasho (DAB-96)	23.42^i–k^	20.0^hi^	258.97^m^	260.35^lm^	22.90^h^	21.06^jk^	268.91^jk^	267.61^k^
CV (%)	5.63	4.97	1.05	1.96	5.81	3.43	1.83	3.43
R^2^ (%)	95.1	96.2	99.7	98.9	93.8	96.7	99.1	96.7
P-value	<0.0001	<0.0001	<0.0001	<0.0001	<0.0001	<0.0001	<0.0001	<0.0001
Eff. to RCBD (%)	95.9	100.2	103.5	91.0	91.1	113.2	103.6	100.6

1 Final PSI were recorded at 75 DAP (2019) and 79 DAP (2020) at Haramaya and 72 DAP (2019) and 77 DAP (2020) at Melkassa; and AUDPC = Area under disease progress curve; CV = Coefficient of variation; R^2^ = Coefficients of determination; and Eff. to RCBD = Efficiency of lattice relative to randomized complete block design. Means followed by the same letter(s) are not significantly different at p < 0.05.

At Haramaya, the final mean ALS severity ranged from 21.88 to 40.24% in 2019, and similarly, it ranged from 18.52 to 37.51% in 2020 cropping season. The highest (40.24%) ALS severity was recorded on the common bean genotype DAB-245, followed by DAB-525 (38.38%) and DAB-379 (33.33%) in 2019 cropping season. The lowest (21.88%) ALS severity was observed on the genotype DRKDDRB-81, followed by the genotype DAB-478 (23.13%) at Haramaya in 2019 cropping season, which was better than the checks Zoasho (DAB-96) and Gorossa (Biofort large seeded-5). Angular leaf spot severity decrease by 50.62% (DRKDDRB-81) and 46.51% (DAB-478) compared to the genotype DAB-245 at Haramaya in 2020 cropping season. A similar trend was also observed regard to final ALS severity at Melkassa both in 2019 and 2020 cropping seasons (Table 4).

### 3.5 Angular leaf spot AUDPC

Analysis of variance of AUDPC showed significant (p < 0.0001) variations among the genotypes for ALS AUDPC at both locations during the 2019 and 2020 cropping seasons. The highest (427.86%-days) AUDPC value was obtained from the genotype DAB-245 followed by DAB-525 (407.81%-days), and DAB-379 (311.24%-days). On the contrary, the lowest AUDPC values were computed for the genotypes DRKDDRB-81 (246.77%-days), DAB-478 (249.98%-days), and the check genotype Zoasho (DAB-96) (258.97%-days) at Haramaya in 2019 cropping season. Likewise, the highest AUDPC values were calculated for the same genotypes (412.89%-days) DAB-245 and (392.06%-days) DAB-525 at Haramaya in 2020 cropping season. However, a comparable reduction of 3.49 and 3.86% AUDPC were computed on genotype DAB-245 and DAB-525 as compared to the 2019 cropping season, respectively at Melkassa. At Melkassa, the same trend was noted as in Haramaya area in 2020 cropping season (Table 4).

### 3.6 Angular leaf spot progression rate (r)

Rapid ALS progression rate (0.043 units day^−1^) was noticed on the genotype DAB-245 at Haramaya testing site in 2019 cropping season. In 2020, as low as 0.093 units day^−1^ ALS progress rate was computed for the same site compared with the fastest disease progress rate recorded on the same genotype in 2019 crop season. A relatively slower ALS progression was calculated for the genotypes DRKDDRB-81 and DAB-478 than for other genotypes evaluated in the study at Haramaya in both 2019 and 2020 main cropping seasons (Table 5). The same phenomenon was noted about ALS progress rate at Melkassa, regardless of the cropping seasons. However, the ALS disease progress rate was relatively higher at Melkassa than at Haramaya in both cropping seasons (Table 5). For example, on the genotype DAB-525 at Melkassa in 2019 cropping season, the disease progressed by 0.0085 units day^−1^faster than at Haramaya site on the same genotype. Similarly, ALS progression rate was 0.001 units day^−1^ faster on the same genotype at Melkassa than at Haramaya site in the 2020 cropping season (Table 5).

**Table 5:** Disease progress rates (units day^−1^) of angular leaf spot (*P. griseola*) on common bean genotypes at Haramaya and Melkassa, Ethiopia, during the 2019 and 2020 main cropping seasons.

Genotype	Progression of ALS at Haramaya^1^						Progression of ALS at Melkassa^1^					
	2019			2020			2019			2020		
	DPR	SE of rate	R^2^ (%)	DPR	SE of rate	R^2^ (%)	DPR	SE of rate	R^2^ (%)	DPR	SE of rate	R^2^ (%)
CCSS6915-11-32	0.025	0.007	93.6	0.021	0.011	86.7	0.025	0.012	95.8	0.028	0.016	92.1
CCSS6915-11-38	0.028	0.020	94.2	0.024	0.013	87.9	0.029	0.015	88.8	0.029	0.019	89.8
DAB-245	0.043	0.011	99.0	0.039	0.018	88.8	0.047	0.021	96.4	0.040	0.026	90.0
DAB-278	0.023	0.008	94.2	0.030	0.013	87.9	0.026	0.015	97.7	0.010	0.019	99.1
DAB-283	0.030	0.007	94.4	0.024	0.011	86.9	0.030	0.012	100	0.026	0.016	89.8
DAB-366	0.026	0.004	98.2	0.018	0.019	92.5	0.036	0.015	90.0	0.022	0.019	93.4
DAB-379	0.034	0.006	94.2	0.027	0.010	95.7	0.041	0.011	98.7	0.030	0.012	94.6
DAB-388	0.027	0.007	97.6	0.022	0.011	93.6	0.029	0.01	99.4	0.029	0.016	96.8
DAB-478	0.012	0.011	89.3	0.010	0.017	84.6	0.013	0.019	98.7	0.012	0.025	95.7
DAB-525	0.036	0.008	94.0	0.039	0.013	86.4	0.044	0.015	97.4	0.037	0.019	96.6
DAB-545	0.022	0.009	91.6	0.020	0.014	90.0	0.022	0.022	99.4	0.022	0.021	94.2
DRKDDRB-65	0.026	0.003	94.1	0.019	0.013	96.9	0.032	0.015	97.8	0.031	0.013	98.6
DRKDDRB-70	0.024	0.006	90.4	0.037	0.010	88.3	0.027	0.011	97.3	0.026	0.017	96.9
DRKDDRB-81	0.010	0.012	93.1	0.009	0.021	89.4	0.011	0.012	94.7	0.010	0.016	95.8
NUA-225	0.019	0.007	94.2	0.016	0.011	96.3	0.021	0.012	97.7	0.021	0.016	94.8
NUA-353	0.024	0.011	90.0	0.016	0.017	98.7	0.031	0.019	96.7	0.027	0.025	99.4
NUA-517	0.021	0.010	96.6	0.020	0.016	94.2	0.034	0.018	95.8	0.023	0.024	97.4
NUA-536	0.026	0.008	93.4	0.022	0.012	87.6	0.033	0.014	97.4	0.013	0.019	96.7
NUA-577	0.023	0.009	94.7	0.023	0.013	90.0	0.028	0.011	93.6	0.017	0.019	98.6
RNSS 6915-89-26	0.032	0.010	99.6	0.026	0.014	90.0	0.031	0.015	98.7	0.032	0.020	94.6
RNSS6915-89-33	0.029	0.014	98.7	0.021	0.018	89.6	0.029	0.020	94.7	0.025	0.026	92.3
SAB-632	0.030	0.006	96.3	0.023	0.010	88.7	0.026	0.011	99.1	0.025	0.024	94.2
SAB-736	0.022	0.008	87.4	0.019	0.020	92.6	0.023	0.015	98.9	0.030	0.019	91.6
Gorossa (Biofort L-5)	0.017	0.005	91.3	0.013	0.012	99.8	0.019	0.013	99.3	0.016	0.013	93.4
Zoasho (RAA-16)	0.015	0.003	93.7	0.012	0.017	98.6	0.017	0.012	99.6	0.013	0.011	92.6

1 ALS = angular leaf spot; DPR = Disease progress rate; SE = Standard error of rate; and R^2^ = Coefficient of determination.

### 3.7 Reaction of common bean genotypes to CBB and ALS diseases

The common bean genotypes evaluated in the study were classified into resistant and moderately resistant reaction groups based on the disease scoring scale assessed at R8 growth stage and the mean disease severity scores assessed during the course of the epidemic period (Tables 6 and 7). At both experimental locations, 21 (84%) common bean genotypes and two checks were regarded as resistant, while 16% (four) of the common bean genotypes exhibited moderately resistant reaction to CBB in 2019 and 2020 cropping seasons. Common bacterial blight mean severity scores ranged from 1.93 to 4.56 at Haramaya and 1.84to 3.77 at Melkassa in 2019 cropping season.

**Table 6:** Reaction groups of common bean genotypes to common bacterial blight (*X. axonopodis* pv. *phaseoli*) and angular leaf spot (*P. griseola*) at Haramaya, Ethiopia, during the 2019 and 2020 main cropping seasons.

Genotype	Reaction of common bean genotypes, 2019^1^						Reaction of common bean genotypes, 2020^1^						
	CBB			ALS			CBB			ALS			
	SS ± SE	Mean	RT	SS ± SE	Mean	RT	SS ± SE	Mean	RT	SS ± SE	Mean	RT	
CCSS6915-11-32	3 ± 0.72	2.17	R	3 ± 2.10	2.77	R	2 ± 1.05	2.70	R	3 ± 1.02	2.19	R	
CCSS6915-11-38	3 ± 0.65	2.12	R	3 ± 1.82	2.62	R	3 ± 0.77	2.49	R	3 ± 1.31	2.21	R	
DAB-245	3 ± 1.06	2.47	R	4 ± 2.17	3.03	MR	3 ± 2.01	2.80	R	4 ± 1.48	3.77	MR	
DAB-278	3 ± 1.27	2.42	R	3 ± 1.96	2.77	R	3 ± 1.54	2.96	R	2 ± 1.27	2.58	R	
DAB-283	4 ± 1.48	3.92	MR	3 ± 2.38	2.96	R	4 ± 1.74	4.92	MR	3 ± 1.27	2.89	R	
DAB-366	3 ± 0.58	2.29	R	3 ± 1.91	2.37	R	3 ± 1.55	2.72	R	3 ± 1.02	2.39	R	
DAB-379	3 ± 0.96	2.07	R	3 ± 1.83	2.62	R	2 ± 1.85	2.83	R	3 ± 1.44	2.40	R	
DAB-388	3 ± 1.10	2.74	R	3 ± 1.95	2.67	R	3 ± 1.09	2.79	R	2 ± 1.16	2.76	R	
DAB-478	3 ± 0.49	2.22	R	2 ± 0.92	2.11	R	3 ± 2.14	2.32	R	3 ± 1.27	2.03	R	
DAB-525	4 ± 1.19	3.36	MR	4 ± 1.61	3.16	MR	4 ± 2.64	3.57	MR	4 ± 1.52	3.59	MR	
DAB-545	3 ± 0.72	1.78	R	3 ± 1.88	2.32	R	2 ± 2.82	2.66	R	2 ± 1.41	2.27	R	
DRKDDRB-65	3 ± 0.79	2.12	R	3 ± 1.73	2.30	R	3 ± 1.97	2.92	R	3 ± 1.45	2.58	R	
DRKDDRB-70	3 ± 1.09	2.62	R	3 ± 1.93	2.15	R	3 ± 2.84	2.12	R	3 ± 1.16	2.76	R	
DRKDDRB-81	3 ± 1.04	2.30	R	2 ± 1.62	1.92	R	2 ± 2.38	2.69	R	3 ± 1.63	1.89	R	
NUA-225	2 ± 1.21	2.62	R	3 ± 1.59	2.22	R	2 ± 1.95	2.52	R	2 ± 1.31	2.21	R	
NUA-353	3 ± 1.06	2.27	R	3 ± 1.70	2.44	R	2 ± 1.74	2.71	R	3 ± 1.02	2.32	R	
NUA-517	3 ± 1.32	2.47	R	3 ± 1.85	2.09	R	3 ± 1.67	2.92	R	3 ± 0.93	2.58	R	
NUA-536	3 ± 0.91	2.16	R	3 ± 1.70	2.00	R	3 ± 1.92	2.72	R	3 ± 1.11	2.30	R	
NUA-577	2 ± 1.01	2.57	R	3 ± 1.92	2.52	R	2 ± 1.90	2.56	R	3 ± 1.31	2.21	R	
RNSS6915-89-26	5 ± 1.71	4.23	MR	3 ± 2.18	2.86	R	6 ± 2.99	4.83	MR	3 ± 1.27	2.93	R	
RNSS6915-89-33	4 ± 1.51	4.56	MR	3 ± 2.17	2.68	R	5 ± 2.94	4.72	MR	3 ± 1.22	2.94	R	
SAB-632	3 ± 0.74	2.52	R	3 ± 1.73	2.44	R	3 ± 1.74	2.41	R	2 ± 1.02	2.21	R	
SAB-736	3 ± 0.72	2.51	R	3 ± 1.35	2.27	R	3 ± 2.11	2.91	R	2 ± 2.01	2.39	R	
Gorossa (Biofort L-5)	2 ± 0.64	2.01	R	2 ± 1.26	1.91	R	3 ± 1.89	2.30	R	2 ± 1.27	2.03	R	
Zoasho (DAB-96)	2 ± 0.73	1.93	R	2 ± 1.23	2.07	R	2 ± 1.86	2.09	R	2 ± 1.16	2.11	R	

1 CBB = Common bacterial blight; ALS = Angular leaf spot; SS = Severity scored at R8 (pod filling stage); and SE = Standard error. Resistance reactions of genotypes were grouped based on the standard severity scale [≤3 (R = resistant); 4–6 (MR = moderately resistant), and ≥7 (S = susceptible)] using van Schoonhoven and Pastor-Corrales (1987) and CIAT (1987). Mean values refer to average of five recordings of disease severity scoring scales at 7 days intervals.

**Table 7:** Reaction groups of common bean genotypes to common bacterial blight (*X. axonopodis* pv. *phaseoli*) and angular leaf spot (*P. griseola*) at Melkassa, Ethiopia, during the 2019 and 2020 main cropping seasons.

Genotype	Reaction of common bean genotypes, 2019^1^						Reaction of common bean genotypes, 2020^1^						
	CBB			ALS			CBB			ALS			
	SS ± SE	Mean	RT	SS ± SE	Mean	RT	SS ± SE	Mean	RT	SS ± SE	Mean	RT	
CCSS6915-11-32	3 ± 1.09	2.29	R	3 ± 1.37	2.85	R	3 ± 1.76	2.21	R	3 ± 1.02	2.24	R	
CCSS6915-11-38	3 ± 0.80	1.95	R	3 ± 1.03	2.86	R	3 ± 0.97	2.36	R	3 ± 1.37	2.54	R	
DAB-245	3 ± 1.48	2.74	R	4 ± 1.65	3.86	MR	3 ± 2.29	2.44	R	4 ± 2.63	3.31	MR	
DAB-278	3 ± 1.24	2.88	R	3 ± 1.29	2.96	R	3 ± 1.94	2.16	R	3 ± 1.66	2.28	R	
DAB-283	5 ± 1.44	3.61	MR	3 ± 1.58	2.96	R	4 ± 1.69	3.13	MR	3 ± 1.33	2.23	R	
DAB-366	3 ± 1.15	2.63	R	3 ± 1.59	2.71	R	3 ± 1.66	2.05	R	3 ± 1.02	2.24	R	
DAB-379	3 ± 1.25	2.98	R	3 ± 1.54	2.88	R	3 ± 1.76	2.05	R	3 ± 1.02	2.27	R	
DAB-388	3 ± 0.88	2.51	R	3 ± 1.64	2.68	R	3 ± 1.86	2.11	R	3 ± 1.04	2.26	R	
DAB-478	3 ± 0.58	2.82	R	3 ± 0.63	2.81	R	3 ± 2.34	2.31	R	3 ± 1.06	2.52	R	
DAB-525	4 ± 1.27	3.54	MR	4 ± 1.87	3.41	MR	5 ± 2.11	3.43	MR	4 ± 2.29	3.71	MR	
DAB-545	3 ± 1.04	2.36	R	3 ± 1.62	2.60	R	2 ± 2.01	2.38	R	3 ± 2.56	2.35	R	
DRKDDRB-65	3 ± 1.32	2.78	R	3 ± 1.37	2.94	R	3 ± 2.07	2.36	R	2 ± 2.11	2.31	R	
DRKDDRB-70	3 ± 1.01	2.67	R	3 ± 1.30	2.81	R	3 ± 1.36	2.15	R	3 ± 2.04	2.26	R	
DRKDDRB-81	3 ± 1.24	2.83	R	3 ± 1.37	2.88	R	2 ± 1.51	2.24	R	2 ± 0.91	1.90	R	
NUA-225	3 ± 0.97	2.95	R	3 ± 1.11	2.67	R	2 ± 1.09	2.19	R	3 ± 2.06	2.28	R	
NUA-353	3 ± 1.25	2.96	R	3 ± 1.16	2.80	R	2 ± 1.76	2.03	R	3 ± 3.02	2.24	R	
NUA-517	3 ± 1.22	2.59	R	3 ± 1.29	2.92	R	3 ± 1.69	2.98	R	3 ± 1.00	2.22	R	
NUA-536	3 ± 1.26	2.90	R	3 ± 1.56	2.83	R	2 ± 1.17	2.06	R	3 ± 0.89	2.18	R	
NUA-577	3 ± 1.22	2.74	R	3 ± 1.10	2.86	R	2 ± 1.97	2.26	R	3 ± 1.45	2.24	R	
RNSS6915-89-26	4 ± 1.30	3.77	MR	3 ± 2.09	2.72	R	5 ± 1.94	3.70	MR	3 ± 2.16	2.28	R	
RNSS6915-89-33	4 ± 1.29	3.52	MR	3 ± 1.65	2.75	R	4 ± 1.07	3.82	MR	3 ± 1.51	2.37	R	
SAB-632	3 ± 0.92	2.35	R	3 ± 1.28	2.77	R	3 ± 1.43	2.01	R	3 ± 2.32	2.14	R	
SAB-736	3 ± 1.08	2.67	R	3 ± 1.25	2.79	R	3 ± 1.23	2.05	R	3 ± 0.94	2.61	R	
Gorossa (Biofort L-5)	3 ± 0.73	2.36	R	3 ± 1.03	2.79	R	2 ± 0.94	2.23	R	2 ± 1.06	2.38	R	
Zoasho (DAB-96)	2 ± 0.56	1.84	R	3 ± 0.87	2.42	R	2 ± 1.86	2.03	R	2 ± 1.74	2.20	R	

1 CBB = Common bacterial blight; ALS = Angular leaf spot; SS = Severity scored at R8 (pod filling stage); and SE = Standard error. Resistance reactions of genotypes were grouped based on the standard severity scale [≤3 (R = resistant); 4–6 (MR = moderately resistant), and ≥7 (S = susceptible)] using van Schoonhoven and Pastor-Corrales (1987) and CIAT (1987). Mean values refer to average of five recordings of disease severity scoring scales at 7 days intervals.

On the other hand, the ALS mean severity scores of 1.92–3.16 at Haramaya and 2.42to 3.86 at Melkassa were recorded in 2019 cropping season (Tables 6 and 7). In addition, mean CBB severity scores ranges 2.09–4.92 at Haramaya and 2.03 to 3.82 at Melkassa were noticed in 2020 cropping season; and the ALS mean severity scores ranged from 1.89 to 3.77 at Haramaya and 1.90 to 3.71 at Melkassa were registered during the epidemic periods of 2020 cropping season. The four moderately resistant genotypes identified were DAB-283, DAB-525, RNSS6915-89-26 and RNSS6915-89-32, which also demonstrated the highest disease severity and AUDPC values and the fastest disease progress rate as compared to other genotypes. Conversely, 23 (92%) of the common bean genotypes including the checks were resistant to ALS both at Haramaya and Melkassa testing sites in the 2019 and 2020 main cropping seasons. However, only two common bean genotypes, namely, DAB-245 and DAB-525 showed moderately resistant reaction to ALS disease both at Haramaya and Melkassa in both cropping seasons though both genotypes had the highest disease parameters. The genotype DAB-525 was founded to react as moderately resistant to both CBB and ALS diseases (Table 7).

### 3.8 Growth and yield components

Days to 50% emergence and stand count at harvest were not significantly (P < 0.05) different among the tested genotypes at both locations in 2019 and 2020 main cropping season. However, days to 50% flowering, days to 90% physiological maturity, plant height, number of pods per plant (NPP), number of seeds per pod (NSP), hundred seed weight (HSW), and grain yield had highly significant (p < 0.0001) variations among genotypes both at Haramaya and Melkassa locations, during the 2019 and 2020 cropping seasons (Tables 8 and 9). The longest mean periods (in days) to 50% flowering were counted on NUA-517 (53.3), NUA-577 (52.6) and SAB-736 (52.3) genotypes at Haramaya in 2019 main cropping season. On the contrary, the genotypes SAB-366D and AB-379 flowered earlier than the other genotypes evaluated at Haramaya in 2019 cropping season. The same phenomena were observed at Haramaya in 2020 cropping season and at Melkassa for common bean growth and yield component parameters in both 2019 and 2020 cropping seasons (Tables 8 and 9).

**Table 8:** Growth performances of common bean genotypes at Haramaya, Ethiopia, during the 2019 and 2020 main cropping seasons.

Genotype	Growth parameters at Haramaya, 2019^1^						Growth parameters at Haramaya, 2020^1^					
	DF	PH	DM	NSP	NPP	SCH	DF	PH	DM	NSP	NPP	SCH
CCSS6915-11-32	45.6^cd^	39.3^de^	89.3^e–i^	5.4^a^	12.1^c–e^	31.5	45.6^de^	37.4^ef^	87.2^e–i^	11.6^c–e^	5.5^a^	31.5
CCSS6915-1-138	44.7^cd^	42.6^cd^	88.3^g–i^	5.6^a^	10.8^ed^	32.8	44.6^de^	33.4^h^	86.4^g–i^	10.4^de^	6.0^a^	32.8
DAB-245	44.6^cd^	37.4^e–g^	92.3^cd^	4.5^bc^	12.5^b–e^	28.2	44.6^de^	35.6^f–h^	90.4^cd^	12.1^c–e^	4.8^ab^	28.2
DAB-278	50.3^b^	41.7^cd^	92.0^cd^	4.0^c^	11.2^ed^	28.8	50.3^c^	39.7^c–e^	90.1^cd^	10.8^de^	3.3^c^	29.0
DAB-283	45.3^cd^	39.2^de^	90.6^d–g^	5.0^ab^	10.9^ed^	27.2	45.3^de^	37.3^ef^	88.7^d–g^	10.5^de^	4.7^ab^	27.0
DAB-366	44.3^cd^	42.7^a–c^	91.3^c–e^	3.8^cd^	12.5^b–e^	28.2	44.3^de^	40.7^a–d^	89.4^c–e^	12.0^c–e^	4.0^b^	28.6
DAB-379	44. 0^cd^	35.8^e–g^	92.0^c–e^	3.5^d^	12.3^b–e^	29.2	40.0^de^	36.6^fg^	90.1^c–e^	11.9^c–e^	3.7^b^	29.7
DAB-388	45.0^cd^	35.1^g^	92.3^cd^	4.0^c^	11.6^ed^	30.2	45.0^de^	40.6^b–d^	90.4^cd^	11.2^de^	3.6^c^	30.4
DAB-478	46.3^b^	38.2^ef^	91.0^c–f^	4.5^bc^	11.1^ed^	33.3	46.3^d^	36.4^fg^	89.1^c–f^	10.7^de^	4.0^b^	33.0
DAB-525	46.0^cd^	43.4^a–c^	88.0^hi^	5.0^a^	12.8^b–d^	29.5	46.0^de^	39.6^de^	86.1^hi^	12.3^cd^	4.5^b^	29.5
DAB-545	45.6^cd^	44.3^a–c^	90.6^d–g^	4.8^b^	10.0^ed^	27.5	45.6^de^	41.3^a–d^	88.7^d–g^	12.2^cd^	4.8^ab^	28.0
DRKDDRB-65	44.6^cd^	38.9^de^	88.6^f–i^	5.3^a^	10.3^ed^	27.5	44.6^de^	37.1^ef^	86.7^f–i^	9.0^e^	6.0^a^	27.5
DRKDDRB-70	50.3^ab^	39.4^de^	90.0^d–h^	5.5^a^	11.3^e^	30.5	50.3^c^	37.5^ef^	88.1^d–h^	9.9^de^	5.2^a^	30.5
DRKDDRB-81	46.0^cd^	37.6^e–g^	88.6^f–i^	5.8^a^	11.8^c–e^	31.2	46.0^de^	35.8^f–h^	86.7^f–i^	11.4^de^	5.5^a^	31.2
NUA-225	51.6^ab^	45.7^a^	96.3^a^	5.5^a^	16.5^b^	30.5	51.3^bc^	43.5^a^	95.4^a^	11.7^c–e^	5.0^a^	30
NUA-353	45.7^cd^	42.9^a–c^	91.3^c–e^	4.5^b^	10.9^ed^	28.8	45.6^de^	40.8^a–d^	89.4^c–e^	10.5^de^	4.8^ab^	28.8
NUA-517	53.3^a^	44.7^ab^	91.3^ab^	6.0^a^	15.0^bc^	28.2	51.4^a^	42.5^a–c^	91.4^ab^	12.7^cd^	5.6^a^	27.8
NUA-536	45.3^cd^	44.8^ab^	90.6^d–g^	4.8^ab^	12.7^b–e^	29.2	45.3^de^	42.2^a–d^	88.7^d–g^	9.7^de^	5.0^a^	29.2
NUA-577	52.6^a^	45.3^ab^	94.3^ab^	5.1^a^	12.8^b–d^	27.5	52.6^ab^	42.6^ab^	95.3^ab^	12.4^cd^	4.8^a^	26.0
RNSS 6915-89-26	45.3^cd^	44.0^a–c^	88.6^f–i^	4.3^bc^	11.2^ed^	29.2	45.3^de^	41.9^a–d^	88.6^f–i^	10.8^de^	4.2^bc^	29.3
RNSS 6915-89-33	46.3^c^	42.6^bc^	88.6^f–i^	4.0^c^	10.9^ed^	28.5	46.3^d^	43.1^ab^	86.7^f–i^	10.6^de^	3.6^c^	28.8
SAB-632	45.0^cd^	35.8^e–g^	89.3^e–i^	4.9^ab^	10.7^ed^	33.1	45.0^de^	34.1^gh^	88.1^e–i^	10.4^de^	5.0^a^	33.4
SAB-736	52.3^ab^	38.4^ef^	87.6^h–j^	4.9^ab^	13.5^b–d^	33.0	52.3^abc^	34.0^gh^	85.7^h–j^	14.6^bc^	4.4^b^	33.0
Gorossa (BL-5)	50.3^b^	39.7^de^	85.6^j^	5.0^a^	13.2^b–d^	33.3	50.3^c^	37.8^de^	84.9^j^	18.7^a^	5.1^a^	33.5
Zoashea (DAB-96)	45.6^cd^	45.2^ab^	85.6^j^	4.0^ab^	19.4^a^	33.5	44.3^de^	42.4^a–d^	84.3^j^	15.8^b^	4.0^b^	33.3
CV (%)	2.48	3.77	1.38	24.8	14.23	8.4	9.36	3.82	2.51	13.44	16.2	29.8
R^2^ (%)	93.1	91.1	90.0	83.1	76.1	61.7	94.1	90.6	77.2	79.1	86.9	0.179
P-value	<.0001	<.0001	<.0001	0.0005	0.0283	0.421	<.0001	<.0001	0.0210	<.0001	0.004	54.7
Eff. to RCBD (%)	102.8	118.2	100.3	97.3	97.2	79.8	104.1	116.2	100.2	101.1	106.6	74.5

1 DF = Days to 50% flowering; PH = Plant height (cm); DPM = Days to 90% physiological maturity; NSP = Number of seed per pod; NPP = Number of pods per plant; and SCH = Stand count at harvest; CV = Coefficient of variation; R^2^ = Coefficients of determination; and Eff. to RCBD = Efficiency of lattice relative to randomized complete block design. Means in a column followed by the same letter(s) are not significantly different at p < 0.05.

**Table 9:** Growth performances of common bean genotypes at Melkassa, Ethiopia, during the 2019 and 2020 main cropping seasons.

Genotype	Growth parameters at Melkassa, 2019^1^						Growth parameters at Melkassa, 2020^1^					
	DF	PH	DM	NSP	NPP	SCH	DF	PH	DM	NSP	NPP	SCH
CCSS6915-11-32	49.0^b–d^	41.7^h–k^	89.3^a–c^	15.2^d–h^	5.4^a–d^	29.8	45.6^c–e^	45.3^b–d^	89.6^ab^	16.9^a–c^	3.5^bc^	30.0
CCSS6915-1-138	47.3^d–f^	45.2^c–h^	89.3^a–c^	16.5^cd^	5.5^a–d^	30.7	44.6^e^	37.2^i^	87.3^a–c^	17.1^a–c^	4.0^b^	31.0
DAB-245	48.6^b–e^	39.4^i–k^	88.6^a–e^	14.4^f–i^	4.7^a–d^	27.3	44.6^e^	52.2^a^	86.3^bc^	9.5^fg^	4.0^b^	27.3
DAB-278	48.3^b–e^	50.1^ab^	89.6^ab^	14.0^h–j^	5.3^a–d^	26.0	45.3^de^	50.8^a^	86.6^bc^	10.7^c–g^	3.7^bc^	26.0
DAB-283	47.0^e–g^	40.9^h–k^	88.3^a–d^	16.0^c–e^	5.0^a–d^	27.0	45.3^de^	40.8^h^	87.3^a–c^	16.6^a–d^	5.0^ab^	27.0
DAB-366	44.6^i^	50.6^ab^	89.6^ab^	14.2^g–j^	5.4^a^	25.5	44.3^e^	46.0^b–d^	87.0^a–c^	9.8^fg^	4.5^ab^	26.0
DAB-379	45.0^hi^	38.1^jk^	89.0^a-d^	15.1^e–h^	4.1^d^	27.5	44.0^e^	36.5^i^	88.3^a–c^	11.7^c–g^	4.0^b^	27.5
DAB-388	47.3^d–f^	37.1^k^	90.0^ab^	14.8^e–i^	5.1^a–d^	25.5	45.0^e^	36.1^i^	88.6^a–c^	8.4^g^	4.6^ab^	25.5
DAB-478	46.0^g–i^	46.5^b–f^	88.6^a–e^	15.6^d–g^	4.9^a–d^	29.8	46.3^c–e^	43.9^f–h^	89.0^a–c^	9.2^fg^	4.6^ab^	30.1
DAB-525	46.0^g–i^	45.2^c–h^	86.3^ef^	13.6^ji^	4.6^a–d^	26.3	46.0^c–e^	50.7^a^	87.0^a–c^	10.7^c–g^	4.0^b^	26.6
DAB-545	46.3^f–h^	47.3^b–f^	88.6^a–e^	13.5^ji^	5.4^a–d^	26.3	45.6^c–e^	41.8^f–h^	87.0^a–c^	11.3^c–g^	4.0^b^	26.0
DRKDDRB-65	47.3^d–f^	42.7^f–j^	88.3^a–e^	14.4^f–i^	5.2^a–d^	28.3	44.6^c–e^	48.1^b^	88.6^a–c^	9.6^fg^	4.3^b^	28.1
DRKDDRB-70	47.3^d–f^	48.2^a–d^	88.6^a–e^	15.7^c–f^	5.2^a^	28.1	50.3^ab^	45.1^c–e^	87.0^a–c^	8.7^g^	4.6^ab^	28.4
DRKDDRB-81	47.0^e–g^	43.3^e–i^	89.0^a–d^	17.0^bc^	5.4^a–d^	29.8	46.0^c–e^	42.5^f–h^	87.3^a–c^	9.1^fg^	5.0^ab^	27.8
NUA-225	46.0^g–i^	51.5^a^	90.5^a^	18.2^ab^	4.4^cd^	29.0	50.0^ab^	52.3^a^	91.0^a^	19.2^ab^	5.2^a^	30.0
NUA-353	49.6^ab^	48.1^a–d^	88.3^a–e^	14.0^h–j^	5.7^a–d^	26.5	45.6^c–e^	50.6^a^	88.3^a–c^	7.8^g^	4.3^b^	26.7
NUA-517	51.0^a^	50.0^ab^	90.1^ab^	17.5^bc^	5.5^a–d^	26.2	52.0^a^	51.9^a^	89.6^ab^	16.5^a–e^	5.0^ab^	26.4
NUA-536	49.0^b–d^	48.2^a–d^	88.9^a–d^	14.4^f–i^	4.8^a–d^	26.7	50.3^ab^	47.1^ab^	88.3^a–c^	11.9^c–g^	4.3^b^	27.0
NUA-577	49.6^ab^	51.3^a^	90.0^ab^	17.1^bc^	4.4^cd^	26.0	52.3^a^	50.6^a^	90.0^ab^	10.8^c–g^	5.0^ab^	26.2
RNSS 6915-89-26	48.6^b–e^	47.5^a–e^	88.3^a–d^	14.5^f–i^	5.4^a–d^	26.5	45.3de	41.5^gh^	86.3^bc^	15.3^a–f^	3.0^c^	26.5
RNSS 6915-89-33	47.6^c–g^	49.1^a–c^	89.6^ab^	16.9^bc^	5.8^a–d^	25.5	46.3^c–e^	37.2^i^	86.0^bc^	10.5^d–g^	5.0^ab^	25.5
SAB-632	49.0^b–d^	38.4^jk^	87.6^a–f^	12.9^j^	5.1^a–d^	28.7	45.0e	37.1^i^	87.3^a–c^	13.4^b–g^	4.0^b^	28.9
SAB-736	49.3^bc^	41.8^g–k^	88.0^a–f^	13.5^ji^	5.0^a–d^	31.3	51.3^a^	42.6^f–h^	89.6^ab^	10.2^fg^	4.6^ab^	31.6
Gorossa (BL-5)	48.0^b–f^	41.3^h–k^	85.6^f^	19.2^a^	5.0^a–d^	32.0	48.2^bc^	44.3^d–f^	85.3^c^	21.0^a^	5.3^a^	32.7
Zoashea (DAB-96)	47.0^e–g^	44.1^d–i^	87.0^c–f^	17.2^bc^	4.4^cd^	31.5	48.0^b–d^	42.9^e–h^	85.3^c^	18.8^ab^	5.5^a^	31.5
CV (%)	1.82	5.54	1.45	4.82	14.8	28.4	11.98	3.08	2.27	20.81	11.7	8.34
R^2^ (%)	87.2	88.3	72.4	91.3	79.7	8.3	88.5	96.7	74.1	77.2	81.4	0.522
P-value	<.0001	<.0001	0.0306	<.0001	0.001	0.334	<.0001	<.0001	0.0149	<.0001	0.003	69.7
Eff. to RCBD (%)	94.7	100	100.5	99.3	104.3	76.5	98.1	88.1	90.8	100	111.5	70.4

1 DF = Days to 50% flowering; PH = Plant height (cm); DPM = Days to 90% physiological maturity; NSP = Number of seed per pod; NPP = Number of pods per plant; and SCH = Stand count at harvest; CV = Coefficient of variation; R^2^ = Coefficients of determination; and Eff. to RCBD = Efficiency of lattice relative to randomized complete block design. Means in a column followed by the same letter(s) are not significantly different at p < 0.05.

At Haramaya, the genotype NUA-225 took the longest days to mature in 2019 (96.3) and 2020 (90.5) main cropping season. On the other hand, check genotypes Gorossa (Biofort large seeded-5) and Zoasho (DAB-96) were found to mature ten and five days earlier than genotype NUA-225 at Haramaya in 2019 and 2020, respectively (Tables 8 and 9). A similar trend was recorded for physiological maturity date at Melkassa in the 2019 and 2020 cropping seasons. The tallest mean plant heights were measured for the genotypes NUA-225 (45.7 cm) and NUA-577 (45.3 cm), while the shortest mean plant heights were recorded on DAB-388 (35.1 cm), DAB-379 (35.8 cm) and SAB-632 (35.8 cm) genotypes at Haramaya in 2019 main cropping season. Variable plant heights were also measured on different genotypes at Haramaya in 2020 cropping season.

The check genotype Gorossa (Biofort large seeded-5) gave the highest NPP, followed by genotype NUA-225 and the check variety Zoasho (DAB-96), and the lowest NPP was recorded from the genotype DRKDDRB-65 at both locations over the two seasons. Regarding NSP, the genotype NUA-517 had higher NSP than the other evaluated genotypes, while DAB-379 recorded the least mean NSP at both experimental sites and seasons. Moreover, HSW ranged from 31.8 to 66.7 g on common bean genotypes SAB-736 and DAB-245 in 2019 cropping season and from 30.4 to 63.9 g on the same genotype in 2020cropping season at Haramaya. Hundred seed weight ranged from 39.9 to 59.1 g and from 36.0 to 56.3 g due to the genotypes SAB-736 and DAB-245 at Melkassa in both 2019 and 2020 cropping seasons, respectively.

### 3.9 Grain yield

The analysis of variance indicated that there a significant (p < 0.0001) difference among evaluated common bean genotypes for grain yield both at Haramaya and Melkassa during the two 2019 and 2020 main cropping seasons (Table 10). The grain yield obtained at Haramaya ranged from 2.26 to 5.05 t ha^−1^ and 1.93 to 4.60 t ha^−1^ at Melkassa in 2019 cropping season. In the 2020 crop year, mean grain yield ranged from 2.75to 4.52 t ha^−1^ at Haramaya and 1.76 to–3.99 t ha^−1^ at Melkassa. About 55.24% yield gap was obtained between the resistant Gorossa (Biofort large seed-5) and the moderately resistant (RNSS6915-89-26) genotypes at Haramaya in the 2019 cropping season. Again, 48.45% grain yield difference was obtained from the genotype Gorossa (Biofort large seed-5) at Haramaya in 2020 cropping season, followed by the genotype NUA-225 (47.64%) compared to RNSS6915-89-26 genotype.

**Table 10:** Mean hundred seed weight (HSW) and grain yield performance of common bean genotypes at Haramaya and Melkassa, Ethiopia during the 2019 and 2020 cropping seasons.

Genotype	Yield parameters at Haramaya^1^				Yield parameters at Melkassa^1^			
	HSW (g)		Grain yield (t ha^−1^)		HSW (g)		Grain yield (t ha^−1^)	
	2019	2020	2019	2020	2019	2020	2019	2020
CCSS6915-11-32	40.2^j^	38.4^k^	2.90^g–j^	3.41^e–i^	45.2^d–h^	48.3^a–d^	3.90^a–d^	2.55^c–e^
CCSS6915-1-138	40.6^j^	38.7^k^	3.08^f–i^	3.24^e–j^	44.9^d–h^	39.7^b–d^	3.43^a–d^	2.66^c–e^
DAB-245	66.7^a^	63.9^a^	2.88^g–j^	2.92^i–k^	59.1^a^	56.3^a^	3.34^b–d^	2.87^b–d^
DAB-278	58.6^b–d^	56.1^bc^	3.86^c–e^	3.35^e–j^	53.3^a–d^	48.3^a–d^	4.12^a–c^	2.83^b–d^
DAB-283	49.4^fg^	47.2^g–i^	2.81^g–j^	2.84^jk^	49.6^a–h^	45.3^a–d^	3.18^b–d^	2.55^c–e^
DAB-366	54.7^de^	52.3^d–f^	3.95^c–e^	3.83^c–e^	49.1^a–h^	52.3^ab^	2.97^dc^	2.24^de^
DAB-379	61.8^b^	59.1^b^	3.23^e–i^	3.16^g–k^	48.0^b–h^	51.0^a–c^	3.36^a–d^	2.46^c–e^
DAB-388	56.4^b–d^	55.3^bd^	3.86^c–e^	3.65^e–g^	56.5^a–c^	51.0^a–c^	3.55^a–d^	2.67^c–e^
DAB-478	53.1^ef^	50.8^e–g^	3.56^d–g^	3.51^e–h^	47.0^c–h^	47.6^a–d^	4.14^a–c^	3.15^a–d^
DAB-525	56.3^b–d^	53.8^c–e^	2.90^g–j^	2.86^i–k^	50.5^a–g^	46.3^a–d^	3.19^b–d^	2.36^de^
DAB-545	59.6^bc^	56.6^ab^	3.07^f–i^	3.01^h–k^	48.5^a–h^	49.0^a–d^	3.51^a–d^	2.48^c–e^
DRKDDRB-65	46.2^g–i^	44.2^ij^	4.05^cd^	2.86^i–k^	42.3^e-h^	41.0^b–d^	3.53^a–d^	2.92^b–d^
DRKDDRB-70	46.5^g–i^	44.5^ij^	3.72^c–f^	3.19^f–k^	42.6^e–h^	41.7^b–d^	3.41^a–d^	2.98^a–d^
DRKDDRB-81	49.0^f–h^	46.9^hi^	3.30^e–i^	3.35^e–j^	41.5^g–h^	40.7^b–d^	3.64^a–d^	2.85^b–d^
NUA-225	61.0^b^	58.4^a^	4.89^ab^	4.45^ab^	58.7^a^	53.0^ab^	4.36^ab^	3.85^a^
NUA-353	61.0^b^	58.4^b^	3.48^d–h^	3.54^e–h^	58.6^a^	50.3^a–c^	3.34^a–d^	2.54^c–e^
NUA-517	53.0^ef^	49.7^f–h^	4.33^bc^	4.05^a–d^	46.9^c-h^	46.6^a–d^	4.30^ab^	3.51^a–c^
NUA-536	59.2^bc^	56.5^bc^	4.06^cd^	3.66^e–g^	51.7^a-g^	46.3^a–d^	4.19^a–c^	3.14^a–d^
NUA-577	58.5^b–d^	56.0^bc^	4.32^bc^	3.97^b–d^	57.9^ab^	51.0^a–c^	4.29^ab^	3.17^a–d^
RNSS 6915-89-26	45.0^i^	43.0^j^	2.26^j^	2.33^k^	48.8^a–h^	46.6^a–d^	1.93^e^	1.76^de^
RNSS 6915-89-33	52.6^ef^	50.4^e-h^	2.78^ij^	2.75^kl^	53.1^a–e^	49.0^a–d^	2.80^d^	2.31^de^
SAB-632	45.1^hi^	43.2^j^	3.68^c–f^	3.73^d–f^	57.2^a–c^	46.6^a–d^	4.16^a–c^	3.12^a–d^
SAB-736	31.8^k^	30.4^l^	2.97^g–i^	3.01^h–k^	39.9^h^	36.0^d^	4.15^a–c^	2.61^c–e^
Gorossa (BL-5)	50.5^f^	48.2^gh^	5.05^a^	4.52^a^	52.7^a–f^	42.6^b–d^	4.60^a^	3.99^a^
Zoasho (DAB-96)	44.3^i^	42.3^j^	4.81^ab^	4.28^a–c^	51.0^a–g^	38.0^cd^	4.35^ab^	3.82^ab^
CV (%)	4.21	3.99	9.91	8.57	10.61	14.39	17.06	18.95
R^2^ (%)	96.7	97.1	89.9	89.5	86.5	74.4	76.5	80.6
P-value	0.0334	<.0001	<.0001	<.0001	0.0045	0.008	0.01	0.0255
Eff. to RCBD (%)	123.5	120.3	94.8	111.6	93.8	102.6	101.8	95.7

1 HSW = Hundred seed weight. CV = Coefficient of variation, R^2^ = Coefficients of determination, and Eff. to RCBD = Efficiency of lattice relative to randomized complete block design. Means in a column followed by the same letter(s) are not significantly different at p < 0.05.

The highest (5.05 and 4.52 t ha^−1^) mean grain yields were obtained from the genotype Gorossa (Biofort large seeded-5) at Haramaya in both 2019 and 2020 cropping seasons, respectively. However, the grain yields obtained were not significantly varied from the mean grain yield obtained from the genotypesNUA-225 (4.89 t ha^−1^), Zoasho (DAB-96) (4.81 t ha^−1^), NUA-517 (4.33 t ha^−1^), NUA-577 (4.32 t ha^−1^)and NUA-536 (4.06 t ha^−1^) in 2019 cropping season and NUA-225 (4.52 t ha^−1^), Zoasho (DAB-96) (4.28 t ha^−1^), NUA-517 (4.05 t ha^−1^) and NUA-577 (3.97 t ha^−1^). The lowest grain yields of 2.26 and2.33 t ha^−1^ were recorded from the genotype RNSS6915-89-26 at Haramaya in 2019 and 2020 cropping seasons, respectively. This genotype poorly performed in grain yield among the evaluated common bean genotypes (Table 10) and suffered more from CBB epidemic development (Tables 2 and 3).

At Melkassa site, the highest mean grain yield of 4.6 t ha^−1^ and 3.99 t ha^−1^ were harvested from the genotypes Gorossa (Biofort large seeded-5) in 2019 and 2020 main cropping seasons, respectively but it was not significantly varied from some of the evaluated common bean genotypes (Table 10). The genotypes Gorossa (Biofort large seeded-5), NUA-517, NUA-225 and Zoasho (DAB-96) maintained consistent grain yield production potential (Table 10). About 58.04and 39.13% grain yield reductions were obtained from the moderately resistant genotypes namely RNSS6915-89-26 and RNSS6915-89-33, respectively, as compared to the resistant genotype Gorossa (Biofort large seeded-5) at Melkassa in 2019 main cropping season. Similarly, about 55.88 and 42.1% grain yield reductions resulted from the moderately resistant genotypes RNSS6915-89-26 and RNSS6915-89-33 in 2020 cropping season as compared to the resistant genotype Gorossa (Biofort large seeded-5), followed by about 54.28 and 40% grain yield reductions were obtained on the same genotypes, RNSS6915-89-26 and RNSS6915-89-33 as compared to NUA-225. The lowest (1.93 t ha^−1^) and (1.73 t ha^−1^) grain yields were obtained from the genotype RNSS695-89-26 at Melkassa in 2019 and 2020 cropping seasons, respectively. Interestingly, the genotype that had a moderately resistant reaction to ALS had the heaviest HSW compared to other reaction groups, regardless of locations and cropping seasons.

### 3.10 Association of grain yield and disease severity

**Figure 3:**
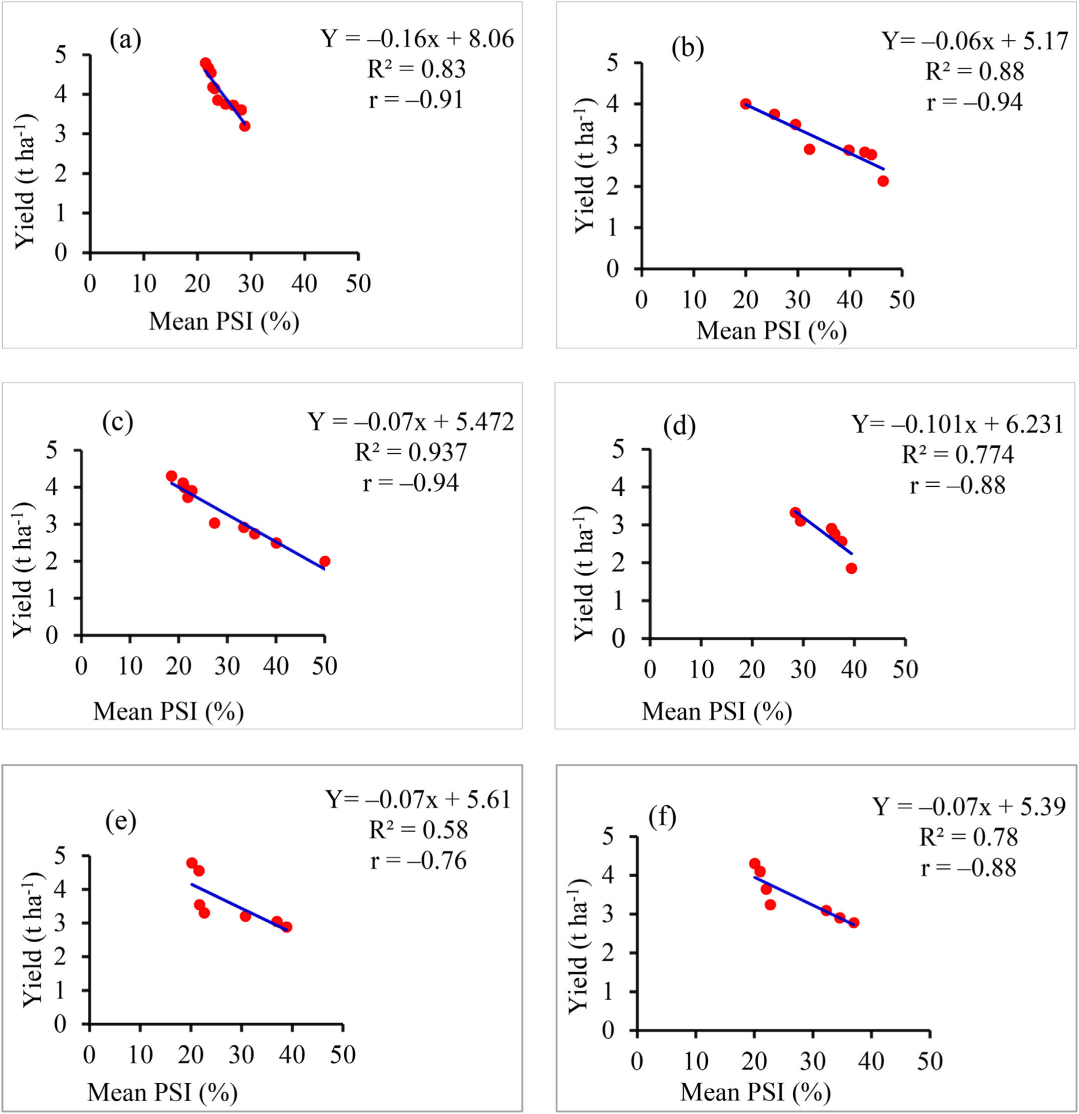
The association of mean PSI (%) of CBB with mean grain yield of CBB resistant group genotypes (a and c) and moderately resistant group genotypes (b and d) at Haramaya and Melkassa, respectively, and the association of overall mean PSI (%) of ALS at Haramaya (e) and Melkassa (f), Ethiopia, during the 2019 and 2020 main cropping seasons.

The associations between mean disease severity and mean grain yields were determined using a simple linear regression model. Variable levels of the associations were established among mean disease severity of different reaction groups of common bean genotypes and their grain yield at both locations in both cropping seasons to CBB and ALS diseases (Figure 3a–f).

The overall association indicated that there was a negative relationship between the disease severity and grain yield (Figure 3). Effects of CBB pressure on the genotypes elucidated that, on average, different reductions can be expected when disease severity increased across locations and main cropping seasons. The linear regression analysis was run to describe the associations between different reactions of common bean grouped genotypes grain yield and CBB disease severity. The mean values of the assessment of CBB mean severity was applied to predict yield reductions per resistance reaction group at Haramaya and Melkassa sites for the 2019 and 2020 cropping seasons (Figure 3a–d).

The average predicted slope of the regression line for CBB resistant genotypes was –0.16 and –0.06 for CBB moderately resistant grouped genotypes at Haramaya was done using the mean CBB severity of 2019 and 2020 main cropping seasons (a and b). Similarly, the slope of –0.07 and –0.101 was predicted at Melkassa for CBB resistant and moderately resistant grouped genotypes, respectively using the mean CBB severity of 2019 and 2020 (c and d). The overall genotypes for both years mean ALS severity and grain yield were applied to predict the yield reduction at Haramaya and Melkassa (e and f). For example, on average, up to 43.4% yield reduction (r = –0.94, R^2^ = 87.7%) can be predicted, if 50% CBB mean final severity is assessed on moderately resistant genotypes and only 1.2% yield reduction can be predicted on CBB resistant genotypes at Haramaya. This implied that when CBB severity rises, it caused a significant reduction in grain yield on moderately resistant common bean genotypes at both locations (a–d). A similar phenomenon can be predicted for ALS disease severity increase at Haramaya and Melkassa.

## 4 Discussion

Remarkable yield loss and worsened food scarcity happened due to plant diseases worldwide (Karavina et al., 2011), and plant diseases are continued to be threat to food security in Ethiopia. Common bean is cultivated in many parts of the world and is the main source of protein, thus; it plays an important role in human diet (Arefeh et al., 2019). Plant diseases are estimated to cause a yield reduction of almost 20% in the principal food and cash crops in the world (Brasier, 2001). For instance, common bean diseases, such as common bacterial blight (Fininsa and Yuen, 2001; Adila et al., 2021) and angular leaf spot (Aytenfsu et al., 2019) are the most widely distributed, severe and devastating common bean diseases in Ethiopia. In the current study, significant (p < 0.0001) variations were observed among the evaluated common bean genotypes to disease reactions and agronomic performances at both locations and during the two cropping seasons.

The findings of the current study revealed that the epidemics due to CBB were slightly higher at Haramaya than at Melkassa in both cropping seasons. This might be due to the variation in altitudinal ranges and associated weather conditions at Haramaya, which could favor epidemic development compared with Melkassa. That is, Haramaya had extended periods of rainy days and a sufficient amount of rainfall (231.5 mm) with cool and warm temperature ranging from 13.5 to 28.5 °C and high relative humidity (>80%) in the crop growing periods (Figure 1). Torres et al. (2009) also reported that favorable weather conditions enhance the growth and rate of reproduction of pathogens and have a discernible effect on the epidemic development. Higher disease pressure due to both CBB and ALS diseases, which might be attributed to the amount of inoculum availability in the soil and crop debris, was recorded at both locations in 2019 than in 2020 main cropping season and marked differences in weather factors between seasons that promote CBB and ALS epidemic development. According to the findings of Emam et al. (2010), cool to warm temperature ranging between (13.5 to 30 °C) and high relative humidity (>70%) favor the active growth of CBB pathogens in the field that resulted from infected seeds and plant debris.

Common bean genotype characteristics and environmental conditions are known to influence and cause variations in plant disease development, either by affecting the host, the pathogen, or even their interactions. For instance, varying weather condition influences the pathogen and host environments in different locations (Dixon, 2012). In addition, angular leaf spot is one of the major common bean diseases in warm and humid areas like Melkassa, where the inoculum sources are supposed to be abundant throughout the cropping season due to all year round circulation of bean cultivation in the area. Hence, warm and slightly humid environmental conditions at Melkassa made slightly higher ALS pressure than the disease intensity at Haramaya. Altitude, daily temperature, rainfall (193.7mm) and relative humidity (>65%) were lower in amount and distribution in the growing periods, which could favor ALS epidemic development at Melkassa more than at Haramaya (Figure 2). It is in favor of the findings of the study by Kimno et al. (2016), who evaluated 34 common bean genotypes with different genetic backgrounds and observed that genotypes attained significant variation with respect to ALS severity. For example, in the report of Zahra et al. (2021), variabilities were seen in pinto, red and white typed common beans for seed morphological characteristics, seed protein percentage, zinc content and mineral nutrients. The outcome variation in locations in the current study could be because of the experiment was performed in open environment and the genotype versus environment interaction was not exactly determined.

Disease severity was used to identify disease resistance reactions and indicated the existence of potential sources of resistance against CBB and ALS diseases in the evaluated common bean genotypes in the two years. Previous studies also reported the presence of marked genetic variation among common bean genotypes with variable reactions to CBB and ALS diseases (Rezene and Mekonin, 2019). Based on low mean disease severity (CIAT, 1987), the present study identified 21 and 23 resistant common bean genotypes including the checks for CBB and ALS, respectively. Accordingly, the nine genotypes DAB-96 (Zoasho), DAB-378, DAB-388, DAB-478, DRKDDRB-70, Gorossa (Biofort large seed-5), NUA-225, NUA-517 and NUA-577 had low mean disease severities and were found resistant to CBB and ALS diseases. In addition, the genotypes CCSS6915-11-32, CCSS6915-11-38, DAB-366, DAB-379, DAB-388, DAB-545, DRKDDRB-65, DRKDDRB-70, NUA-353, NUA-536 and SAB-736 also exhibited resistance to CBB and ALS diseases.

Variable reactions among the genotypes showed the possibility of obtaining genes for resistance and confirmed the presence of genes for resistance having varying genetic potentials to adjust under different environments (Rezene and Mekonin, 2019; Tumsa et al., 2020; Adila et al., 2021). Beebe et al. (2010) indicated a wider window for variation in phenology and disease resistance ability could be due to enormous variation in the gene pools and races of genotypes. Hence, it could be evidence to get sources of disease resistance from elite lines, landraces and released varieties, probably also from gene pools could be used in the common bean breeding system. In this regard, studies by Tumsa et al. (2020) tested 110 bean genotypes and found that 32 of the genotypes had superior CBB resistance reaction and agronomic performances which confirm for the present finding. Similarly, Adila et al. (2021) reported that common bean genotypes performed variably to CBB infections, grain yield and yield components under field conditions. Interestingly, only genotypes NUA-517, NUA-225, and NUA-577 are more comparable to both checks in CBB disease resistance in both locations and both years. On the other hand, more than 90% of the common bean genotypes were resistant and only below 10% of the genotypes were moderately resistant to ALS at both locations in the two seasons. In fact, for ALS disease there are several genotypes perform similar to both checks, but grippingly, DAB-478 is the best genotype, next to checks, that performed very well to ALS disease resistance in both locations and seasons. However, only the genotypes DAB-245 and DAB-525 were moderately resistant to ALS, while most of the genotypes exhibited resistant reactions.

Of course, the previous common bean accessions collected across Ethiopia demonstrated varied resistance reactions to ALS disease under natural infections (Rezene and Mekonin, 2019). This could imply that the probable availability of sources of disease resistance genes in common bean genotypes in the country, and diseases resistance disparities among genotypes can be the best option to manage ALS disease. Moreover, development of common bean cultivars with disease resistance is one of the main aims of common bean breeding programs, as continuous emergency and distribution of new ALS races in the country (Rezene et al., 2018) could break down disease resistance in common bean genotypes. Hess et al. (2002) also concluded that genetic resistance obtained from source materials has been considered as the most desired approach for successful plant disease management. In this connection, the present study confirmed that the check genotypes Zoasho (DAB-96) and Gorossa (Biofort large seeded-5) and DAB-388, DAB-478, DRKDDRB-81, NUA-225, NUA-517, NUA-577 and SAB-632 were highly resistant to both diseases and indicated good agronomic performances across locations and over seasons.

Growth and yield components can be significantly influenced by seed traits, like seed size and color, and environmental factors that finally impose impacts on the proper physiological and grain filling process on a given plant stands (Wang et al., 2016; Moosavi et al., 2022). In the present study, on the basis of growth, yield and yield component performances, genotypes, such as DAB-388, NUA-225, NUA-536, NUA-517and NUA-577were promising to get better agronomic advantages at different agro-ecologies next to the checks. Such genotypes could serve as sources of resistance genes to incorporate into cultivars having desirable agronomic characters; and some of the genotypes could be released as varieties since they showed good yielding potential, desirable morphological characters and growth habits. For example, the genotypes DAB-388, NUA-517, NUA-225 and NUA-577 were resistant to CBB and ALS and had high yielding potential and that can be used for production in relatively high rainfall receiving areas, sub-humid areas of eastern Ethiopia for different cropping systems and the Central Rift Valley parts of the country. The identified genotypes obtained equivalent yield advantages to the currently available common bean variety, such as Zoasho (DAB-96) and other cultivated varieties. The high yielding potentials of the identified genotypes compared to the checks at the study sites were likely to be due to their resistance response to multiple diseases and the genetic background of the genotypes. In general, the responses of genotypes to CBB and ALS diseases variably, showed there was possibility of obtaining genes for resistance and it confirmed that the presence of resistant genes in the donor parents. This could have an implication of the potential to develop resistant varieties with acceptable agronomic traits in the existing land races of common bean in the country.

The overall performance of the common bean genotypes, in mean grain yield, relatively performed better at Haramaya site (3.73 t ha^−1^) than at Melkassa (3.23 t ha^−1^). This could be attributed to the adaptation of the genotypes to the sub-humid highlands of Haramaya and suitability for other environmental factors. The following genotypes: DAB-388, Gorossa (Biofort large seed-5), NUA-517, NUA-225, NUA-536, NUA-577, and Zoasho (DAB-96), were identified for better agronomic performance regardless of locations and seasons. These genotypes maintained higher values of number of pods per plant, number of seeds per pod and grain yield. These identified complementary genotypes are promising genotypes for direct production. Adila et al. (2021) indicated that common bean genotypes responded variably to yield and yield related parameters studied for CBB at southern parts of Ethiopia. In Arefeh et al. (2019) experiment, it was observed that the agro-morphological and biochemical traits of white bean were affected by various factors like hormonal effects and the condition resulted variation effect on the crop traits. Similarly, Nene, 1998 in his first report indicated that, growing common bean genotypes possessing multiple disease resistance minimizes yield losses, reduces the need for chemicals and lowers production costs. In another study, as indicated by Moosavi et al. (2022) and Marcos (2015), seed formation and grain filling in castor beans might be affected by seed size and color and result in lower and less dense and poor vigor seed. Therefore, the lastly produced grains lose ideal grain filling conditions, which cause them to be smaller, lower seed weight and have low vigor and viability.

Reliable predictions of the impact of diseases on yields are prerequisites to establish any disease management strategies (Gaunt, 1995), and thus, crop yield loss assessment is considered as an important component in disease management studies to improve crop production for smallholder farmers and ensure food security worldwide (Cerda et al., 2017). In this current study, despite the genotypes showed potentially advantageous grain yield production, disease severity and grain yield were established significantly negative relationship at both locations in both years. As a result, some yield reductions were predicted on the different resistance reaction groups of the genotypes due to CBB and ALS disease. This might explain that disease pressure had negative effects on the crop developmental stages. In common bean genotypes evaluation studied by Lemessa and Tesfaye (2005), angular leaf spot severity negatively correlated with yield parameters and, similarly, Tumsa et al. (2020) and Adila et al. (2021) reported that, CBB disease parameters and agronomic traits were negatively correlated. A yield reduction prediction analysis indicated that, there was a negative relationship between disease severity and grain yield, especially on moderately resistant genotypes to CBB, both at Haramaya and Melkassa, clearly suggested that disease severity had adverse impacts on grain yields.

## 5 Conclusions

In the study it was investigated that significant variation observed in CBB and ALS diseases resistance, growth, grain yield and yield component performance among the 25 common bean genotypes evaluated at Haramaya and Melkassa in the 2019 and 2020 main cropping seasons. The study revealed that CBB and ALS were the most important and dominant diseases occurred on the evaluated common bean genotypes. Some common bean genotypes were found as potential sources for disease resistance and better agronomic performances; could serve to develop superior high-yielding and disease resistant varieties. The six genotypes, namely, DAB-366, DAB-388, NUA-225, NUA-517, NUA-536 and NUA-577 along with the two checks could be recommendable for high grain yield as well as sources of resistance to CBB and ALS diseases to use them with proper and recommended agronomic practices. However, the remaining 13 genotypes also had relatively resistant reactions to CBB and ALS diseases, recommended for production after verification across agro-ecologies where CBB and ALS diseases are widely distributed. A negative association was established between CBB and ALS diseases severities and grain yield of the various reaction groups of common bean genotypes. It is suggested that a large number of common bean accessions should be evaluated in CBB and ALS hot spot agro-ecologies of Ethiopia for more sources of resistance and better agronomic advantages. Generally, to assist resource poor farmers by augmenting their incomes, plant breeders and pathologists must give priority to ensure stability of well adapted and popular local landrace common bean for sustainable production and productivity through incorporating multiple disease resistance into cultivated varieties.

## Declarations

### Author contribution statement

Fekede Girma: Conceived and designed the experiment; Performed the experiments; Analyzed and interpreted the data; Wrote the paper.

Chemeda Fininsa: Conceived and designed the experiment; Wrote the paper.

Habtamu Terefe: Conceived and designed the experiment; Analyzed and interpreted the data; Wrote the paper.

Berhanu Amsalu: Contributed reagents, materials, analysis tools or data; Wrote the paper.

### Funding statement

This research did not receive any specific grant from funding agencies in the public, commercial, or not-for-profit sectors.

### Data availability statement

Data will be made available on request.

### Declaration of interest's statement

The authors declare no conflict of interest.

### Additional information

No additional information is available for this paper.
